# High-resolution simulation and validation of soil moisture in the arid region of Northwest China

**DOI:** 10.1038/s41598-019-52923-x

**Published:** 2019-11-21

**Authors:** Xianyong Meng, Hao Wang, Ji Chen, Mingxiang Yang, Zhihua Pan

**Affiliations:** 1 College of Resources and Environmental Science, China Agricultural University (CAU), Beijing, 100094 China; 2 Department of Civil Engineering, The University of Hong Kong (HKU), Pokfulam, 999077 Hong Kong China; 3 State Key Laboratory of Simulation and Regulation of Water Cycle in River Basin & China Institute of Water Resources and Hydropower Research (IWHR), Beijing, 100038 China

**Keywords:** Hydrology, Atmospheric dynamics

## Abstract

Soil moisture plays an important role in land-atmosphere interactions, agricultural drought monitoring, and water resource management, particularly across arid regions. However, it is challenging to simulate soil moisture of high spatial resolution and to evaluate soil moisture at fine spatial resolution in arid regions in Northwest China due to considerable uncertainties in forcing data and limited *in situ* measurements. Then, the data set was used to produce the 1 km high-resolution atmospheric forcing datasets and to drive the Community Land Model version 3.5 (CLM3.5) for simulating spatiotemporally continuous surface soil moisture. The capabilities of soil moisture simulation using CLM3.5 forced by the XJLDAS-driven field were validated against data obtained at three soil layers (0–10, 0–20, and 0–50 cm) from 54 soil moisture stations in Xinjiang. Results show that the simulated soil moisture agreed well with the observations [CORR > 0.952], and the intra-annual soil moisture in Xinjiang gradually increased during May through August. The main factors that affect changes in soil moisture across the study region were precipitation and snowmelt. The overall finding of this study is that an XJLDAS, high-resolution forcing data driven CLM3.5 can be used to generate accurate and continuous soil moisture of high resolution (1km) in Xinjiang. This study can help understand the spatiotemporal features of the soil moisture, and provide important input for hydrological studies and agricultural water resources management over the arid region.

## Introduction

Soil moisture, a key component of terrestrial surface processes, has an important impact on multiple research fields including regional climate analyses, hydrological forecasting for watersheds, meteorological forecasts, and drought monitoring and forecasting for agriculture and forestry industries^1–6^. Soil moisture can also affect the latent and thermal heat transfer of the terrestrial surface by changing the soil heat flux, surface albedo, and other parameters related to the underlying surface, thus further affecting the regional climate^7^. Among the various factors that influence the energy fluxes in the earth system, soil moisture is just after the ocean temperature^8,9^. However, the simulation of soil moisture levels (SMLs) associated with hydrological processes of the terrestrial surface is always challenging because the SML changes are mainly the cumulative effects of various hydrological components and are also affected by other factors such as solar radiation, evaporation, and soil and atmospheric temperatures^10^. Evapotranspiration provides and important feedback on soil moisture, terrestrial (atmospheric) temperatures, and other factors^3,9,11–15^. Water is the source of life, thus soil moisture has a great impact on the vegetation cover. The survival and growth of vegetation cover are inseparable from surrounding living conditions (such as SMLs and soil temperatures).

With the advancement of atmospheric science and research on the terrestrial surface processes, many researchers around the world have focused on soil moisture studies in recent years. However, soil moisture data remain scarce for some reasons such as the limited number of observation stations, limited measurement history, and the cost^16^. At present, published studies based on observation stations have mostly been focusing on the characteristics of SML changes at singular points or in small areas, and it is difficult to conduct climatological studies on SMLs at the global or continental scale^17^. In recent years, significant advancements have been made with respect to the observation of soil moisture via satellite remote sensing, especially in terms of global coverage. Although soil moisture data can be extracted from remote sensing data at various resolutions, the observations are indirect. The existence of substantial uncertainties in remote sensing data further prevented the acquisition of ideal inversion results^18^. There is still a lack of soil moisture data for China and the world at the finer spatiotemporal scale due to the limited number of observation stations and low frequency of measurement. The distribution of observation stations in Northwest China is particularly scarce, especially with respect to soil moisture observation stations^19^.

Many researchers have chosen to use the hydrological migration module of various land models to simulate SMLs in China. Some researchers consider that using land models for numerical simulations may be an effective approach to study SML changes. For example, Du *et al*.^20^ made use of reanalysis data for the period of 1979–2003 provided by the United States Department of Energy (DOE) and the National Centers for Environmental Prediction (NCEP) of the National Oceanic and Atmospheric Administration (NOAA)and the CLM Version 3.5 (CLM3.5) to simulate SMLs in China. They found that the SMLs became more steady with the increase in soil depth. After utilising data from meteorological stations to drive the CLM3.5 for a long-term simulation of the spatiotemporal distribution of soil moisture in China, Li *et al*.^21^ discovered that the model accurately reflects the distributional characteristics. Huang *et al*.^12^ employed Huai-He River Basin Experiment (HUCEX) data to verify the performance capabilities of land models. Chen *et al*.^22^ and Xiong *et al*.^23^ conducted studies on SMLs and soil temperatures in China, respectively. These were macroscopic evaluations on a regional scale. Both studies concluded that the spatiotemporal distribution of China’s soil temperatures and SMLs can be reproduced by using driving data to force land models.

The use of land models for simulations of spatiotemporal SML changes research in China by researchers promoted better SML analyses. However, when data with coarse resolution were used for modelling, the results did not reflect details on the change process within the study area. This is especially the case for watersheds, which are relatively small in scale but contain terrestrial surface elements with vast spatiotemporal variations. It is well known that the accuracy of the land modelling results is determined to a large extent by the atmospheric forcing datasets^24^. When simulations based on land models were conducted using data from China’s relatively scarce stations or reanalysis data with coarser resolution from overseas, the results failed to describe the evolutionary processes that terrestrial surface components within the watersheds undergo in detail. Furthermore, the introduction of low-resolution atmospheric forcing datasets to a model resulted in a significant increase in the amount of uncertainties, which greatly reduced the reliability of the model output. There is an urgent need for reliable and high-precision atmospheric forcing datasets that can drive land models for China’s Xinjiang Region with scarce observation stations and vast spatiotemporal differences. When such an atmospheric forcing datasets is used to force CLM3.5, the continuous evolutionary processes of related terrestrial surface components (such as snowmelt and SMLs) in Xinjiang can be properly simulated.

The Xinjiang Uyghur Autonomous Region of China is located in the hinterland of the Eurasian continent. Its unique topography, comprising three mountain ranges interspersed by two basins, leads to vast spatiotemporal variations in the regional climate. Among the crisscrossing mountain ranges, seasonal permafrost, snow, and glaciers play important roles in maintaining the region’s water supply sources, and thus they make the region’s distribution of water resources extremely complex. There is a lack of atmospheric observation stations and simulations of the region’s hydrological and ecological processes. Consequently, the understanding of the spatiotemporal distribution of terrestrial surface components is extremely limited. Being important indicators for snowmelt, SMLs can be used to accurately determine the infiltration volume of snowmelt. Concurrently, details of the regional climate in which the watershed is situated, such as the terrestrial surface and atmospheric temperatures, and vegetation coverage, can be identified^10^. The aforementioned studies highlight the significance of soil moisture studies for both the relevant academic disciplines and soil moisture actual evolutionary processes Research in Xinjiang. Such studies also help researchers from related fields to further understand terrestrial surface–atmosphere interactions.

This study used two steps to address the lack of high-resolution historical datasets on SMLs in West China and the urgent need for a high-precision atmospheric forcing datasets for the region. First, a 1 km high-resolution atmospheric forcing datasets for Xinjiang (XJLDAS) was created using the assimilation technique. It was specifically tailored for the region and the first of its kind internationally. Next, the XJLDAS was employed to drive the CLM3.5 for simulations of the region at an hourly resolution. In addition, the ecologically fragile watershed of the Bortala and Jing rivers was selected as typical study area to analyse the spatiotemporal responses of terrestrial surface components. The aim was to verify the following: (i) causes of SML changes in the region and patterns of their spatiotemporal evolution, and (ii) usability of the XJLDAS dataset for Xinjiang.

## Methods

### Introduction to the study area

**Figure 1:**
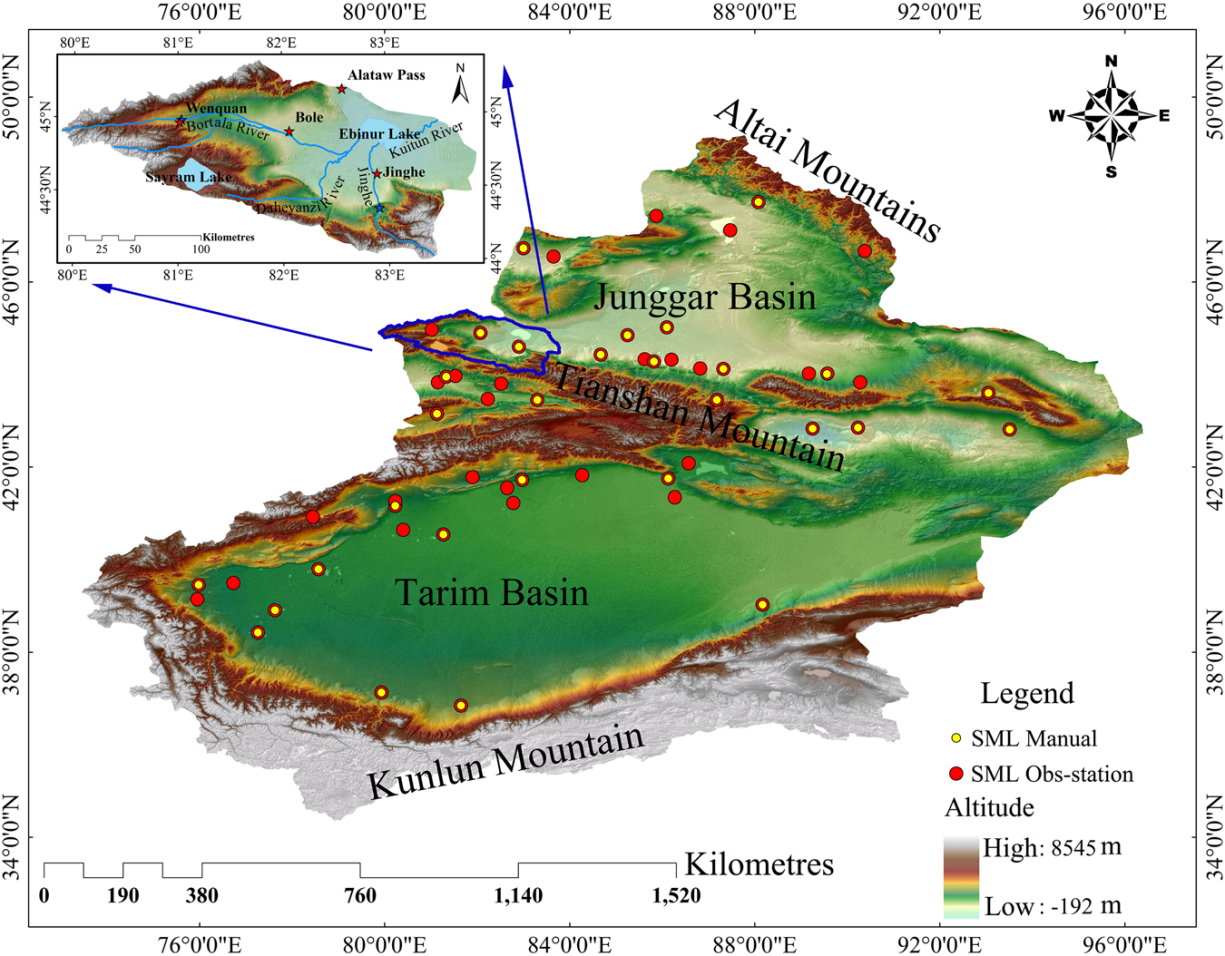
The location and elevation of and the SML stations in the Xinjiang Region and Bortala–Jing Rivers Basin.The map was generated with ArcMap Version 10.1 (http://www.esri.com/en/arcgis/arcgis-for-desktop/).

The Xinjiang Uyghur Autonomous Region of China is located between 73°40′E–96°18′E and 34°25′N–48°10′N (Fig. 1). It has a total land area of 1,664,900 km^2^. Within the region, two basins/valleys are interspersed and surrounded by three mountain ranges, with the Altai Mountains and Kunlun Mountain Range located to the north and south, respectively. Such a unique topography caused extremely complex conditions of the region’s underlying surface and created vast spatiotemporal differences in the climatic distribution. Sunshine is abundant (2,500–3,500 hours per annum). However, regional differences in temperatures are large, with overall temperatures in the south being higher than that in the north. The Bortala–Jing River Basin (area bounded by the red line in Fig. 1) is located on the northern slope of the West Tian Mountains (Tianshan) with an area of 11,275 km^2^. In recent years, the ecological environment experienced accelerated degradation, raising concerns among researchers.

The basin is characterized by more mountainous areas than plains and more north- than south-facing slopes, and the western portion of the basin is even more mountainous than the eastern one. Water vapour from the Arctic and Atlantic oceans serves as the main source of precipitation. Nearly 460 glaciers of various sizes are distributed within the watershed. The total glacial reserves cover 15.4 km^2^, making these regional glaciers the main source of runoff recharge. The glacial area of the surrounding regions of the Jing and Bortala rivers is 96.2 and 110.3 km^2^, respectively. The annual glacial supply to the two rivers is approximately 96 and 105 mio m^3^, respectively, accounting for 20.6% and 21.4% of the respective total river runoff mio m^3^. The runoff of the region’s rivers is mostly recharged by ice, snow, rainfall, and groundwater due to the combined effects of temperature, precipitation, terrain, and other factors.

Recent studies of the region around the Bortala and Jing rivers and the entire Xinjiang Region by Chinese scholars have been based solely on reanalysis data (or regional climate models) and data observed at singular points. So far, high-resolution and -quality simulations and analyses of SMLs using land models have not been carried out due to relatively scarce meteorological stations in Xinjiang. When atmospheric forcing datasets based on data with coarser spatiotemporal resolution are used, the reliability of the simulation results will be severely impacted. It is thus imperative to prepare atmospheric forcing datasets with high spatiotemporal resolution for Xinjiang and then use them to carry out hourly high-resolution simulations and research on the region’s SMLs and other terrestrial surface components.

### Preparation and validation of the XJLDAS dataset

In this study, a 1 km high-precision atmospheric forcing datasets was created for Xinjiang. The parameters of the XJLDAS dataset, such as temperature, pressure, specific humidity, and wind velocity, were assimilated using the Local Analysis and Prediction System (LAPS)/Space and Time Mesoscale Analysis System (STMAS). The LAPS/STMAS is a new-generation data assimilation system that supports the integration of conventional data and various types of detection data (including that from satellites, radar, and automatic stations). After the analysis, the system generates a dataset with high spatiotemporal resolution, which approximates actual observations. This assimilation system was designed to improve the accuracy of conventional forecasting and analysis.

Concurrently, STMAS was specifically designed to improve the Ensemble Kalman filter (EnKF)/four-dimensional variational data assimilation method (4D-Var). Using the multigrid variational technique^25^ and advantages of EnKF/4D-Var, the simulation defects of previous models were eventually reduced by STMAS. Compared with the conventional LAPS, the module for terrestrial surface analysis in the LAPS of the new-generation system was replaced by the corresponding module in STMAS, while the modules for temperature and wind velocity were replaced with the STMAS3D module. The prototypes for STMAS3D/4D analyses have already been widely adopted by NOAA, Federal Aviation Administration (FAA), and other units. At present, the STMAS 3D/4D module can be used to analyse all observed data, radar radial wind fields, and Stepped Frequency Microwave Radiometer(SFMR) data. For Weather Research and Forecasting (WRF) Model, the STMAS analysis field is used as the initial forecasting field for local hurricanes.

#### Core algorithm of the STMAS module within the LAPS framework

The STMAS was proposed by Dr. Xie, a research scientist of NOAA. Although it is an algorithm based on the LAPS framework, it differs from LAPS because it incorporates the multigrid variational technique. Additionally, the STMAS module for terrestrial surface analysis does not make use of the corresponding module in LAPS.

For multigrid variants, the objective function of each grid is shown in Eq. 1^25^:

1 $${J}^{(n)}=\frac{1}{2}{X}^{(n)T}{X}^{(n)}+\frac{1}{2}{({H}^{(n)}{X}^{(n)}-{Y}^{n})}^{T}{O}^{(n)-1}({H}^{(n)}{X}^{(n)}-{Y}^{(n)})\,(n=1,2,3\ldots ,N)$$

where *O* is the covariance of errors of the observed data; *X* represents the modified vector, which can be generated using a variational system (*X* = *X*_*a*_ − *X*_*b*_); *Y* is the difference between the model and measured fields (*Y* = *Y*_*obs*_ − *HX*_*b*_); *Y*_*obs*_ is the measured vector; *X*_*a*_ and *X*_*b*_ represent the vector of the analysis and background fields, respectively; *H* is the bilinear interpolation operator used for the interpolation of the model results into the observed grid; *n* is the number of grids in the *n*^*th*^ layer; and *N* is the multiplicity of the grid^25^.

2 $$\{\begin{array}{ll}{Y}^{(1)}={Y}^{obs}-{H}^{(1)}{X}^{b} & (n=1)\\ {Y}^{(n)}={Y}^{(n-1)}-{H}^{(n-1)}{X}^{(n-1)} & (n=2,3,\cdots ,N)\end{array}$$

During analysis, the multigrid variational technique (STMAS) complies with the rule of going from broad to fine. Starting with a broader grid opening, when the value of *n* is 1, *Y*^(1)^ is the bias between the measured and model estimation fields after these have been mapped onto the measured position. After the operation for *J*^(*n*−1)^ is completed, *X*^(*n*−1)^ is interpolated into the finer grids in the *n*^*th*^ layer. Eventually, the analysis of grids becomes increasingly fine. At the same time, long and short waves are used to test the measured data to create observed data with different resolutions and at various scales^25^. During the aforementioned multiscalar analysis, the results observed at different scales are consistent with the covariance matrix *O*^(*n*)^ of the corresponding observed errors.

Eventually, the multigrid variants are superimposed to^25^:

3 $${X}^{a}={X}^{b}+{X}_{L}={X}^{b}+\mathop{\sum }\limits_{n=1}^{N}{X}^{(n)}$$

The multigrid variational technique differs from three-dimensional variational assimilation (3D-Var) in that the latter often confuses information about long and short waves from observation data. Therefore, the analysis results contain more errors. Given the temporal and spatial distribution of meteorological data is uneven for Xinjiang, the issue of errors is further worsened. On the other hand, the use of the multigrid variational technique to assimilate the 1 km atmospheric forcing datasets (XJLDAS) ensured higher accuracy of the offline data for subsequent input into the CLM3.5 at a later stage of the study.

#### Assimilation of input data

To ensure the resolution and reliability of post-assimilation data, this study employed the LAPS technique to assimilate multivariate data and revise the background field. The assimilation process incorporated a series of data from national automatic stations, regional encrypted stations, and conventional ground stations, sounding data, and radar data. Several important types of input data are described below.

- Data observed at ground stations: Related data were provided by 105 national automatic meteorological stations and ~40,000 regional automatic stations, which have been set up in Xinjiang. These data were subject to strict quality control.
- reanalysis products: The STMAS background field uses 6-hourly data on pressure, potential temperature, and vorticity for regional models from the ERA-interim dataset published by the European Centre for Medium-Range Weather Forecasts (ECWMF). The data products were released by the Integrated Forecasting System (IFS) system established by the ECWMF in 2006. This system contains a 3D-Var module for 12-hourly analysis windows.

#### Assimilation of the data of the XJLDAS dataset

Assimilation of the parameters of the XJLDAS dataset involved multiple analytical processes including that for wind analysis, ground analysis, temperature analysis, clouds analysis, water vapour analysis, diagnostic information analysis and soil temperature analysis. The first module was mainly used for system analysis of high-altitude, three-dimensional wind fields. Two-dimensional analyses of temperatures, pressures, humidities, and wind velocities at the terrestrial surface were carried out with the second module. High-altitude temperature fields were analysed with the third module, while three-dimensional analyses of clouds were conducted with the fourth module. Lastly, the fifth module used observed water vapour data to correct biases generated by the first four modules.

In the LAPS system, the aforementioned analyses must be carried out sequentially and in the right order because the analytical results of a precedent module serve as input for the analysis by an antecedent module. After all modules completed their analyses, the final output is used for weather diagnosis. This study made use of the STMAS embedded in the LAPS system, which allowed the integration of the advantages of other methods (such as Barnes Analysis and EnKF). Therefore, conventional objective analyses could be simulated and appropriate physical equilibria and dynamic descriptions could be adopted for various analyses.

#### Establishing the precipitation and radiation parameters of the XJLDAS dataset

The precipitation data in the XJLDAS dataset were based on precipitation grid products of the China Meteorological Administration (CMA) and were subjected to bilinear interpolation and extraction. The raw data included hourly precipitation data for China prepared by the National Meteorological Center (NMC) of the CMA, hourly precipitation from Yengyun-2ESatellite (FY-2E) and other geostationary satellites, and CPC MORPHING Technique (CMORPH) satellite fusion precipitation products from the NCEP Climate Prediction Center (CPC). A two-step method involving probability density function (PDF) and optimal interpolation (OI) was used for data integration. The product resolution of 0.1° × 0.1° met the accuracy requirements of this study. The radiation data of the XJLDAS dataset were acquired through interpolation of CLDAS2.0 data. The data source for the latter was the inversion of shortwave solar radiation data of Grade 1 products from the meteorological satellites FY2C\E\F in geostationary orbits.

The effects of the elevation and topography were fully considered using cokriging methods during the interpolation of precipitation and radiation parameters to avoid the introduction of false information. The sources of both types of raw data were CMA products. Furthermore, CMA assessed the aforementioned variables within China’s territories and obtained excellent evaluation indicators^1,24^. As such, this study did not carry out further or specific evaluations of radiation and precipitation parameters.

### Brief description of CLM3.5

The CLM3.5 model was developed by numerous scientists and researchers who made use of various methods to combine numerical climatic, ecological, and hydrological simulations into the datasets. CLM3.5 includes the advantages of different types of internationally used land models such as LSM^26^, IAP94^27^, and BATS^28–30^. The CLM is one of the most reliable current models in terms of its capabilities simulating terrestrial surface processes. So far, the CLM series model has been updated to the CLM4.5 version. The CLM4.0 version mainly corrects the numerical solution of the Richards equation, replaces the resistance coefficient with the soil evaporation resistance function, improves the soil boundary conditions, and is the direct coupling of groundwater and soil water. This version also takes into account the influence of the internal stability of the canopy litter and Crown layer, the influence of soil organic matter on the water movement, etc^31^.

The CLM4.5 model has been integrated as a terrestrial component in the Earth System Model CESM1.2.0 released by NCAR, which improves vegetation radiation processes and related parameters^32^, an optional hydrological process has also been added to VIC^32,33^.

### Replacement of a terrestrial surface parameter set (surfdata) with 1 km resolution CLM3.5 data

One of the focus of this study was surfdata substitution and refinement to match the resolution of the atmospheric input data. In recent years, many scientists studied the impact of surfdata substitution on the quality of land model outputs^34–37^. Presently, parameters related to the terrestrial surface that can be substituted include soil texture and colour, land use, and lakes and glaciers. Among these, the soil texture can greatly affect relevant physical parameters such as the hydraulic conductivity coefficient, soil thermal capacity, and saturation of soil water content. On the other hand, terrestrial surface coverage can affect the surface albedo, thereby changing the terrestrial flux equilibrium and water cycle. Scientists recognised as early as the 1980s that land cover types affect the climate differently. For a series of land models, they also considered the role vegetation cover plays in terrestrial water and energy cycles^28,38–42^. A comparison was made between the findings of earlier researchers and requirements of this study to refine and substitute the raw surfdata for CLM3.5. After analysing the various types of raw data included in the model, the data resolution was quite coarse (Supplementary Fig. S1-2 online, left column). The model’s raw data on soil texture^43^, with a resolution of 5 min, were derived through interpolation of global data developed by the Food and Agriculture Organisation of the United Nations (FAO)using 61 of China’s soil profiles. The soil colour parameter was proposed by Zeng *et al*.^44^ who derived the data by integrating satellite data with that of Dickinson *et al*.^40^. The percentages of wetlands and lakes were obtained using multi-year data on fresh water and swamps by Cogley^45^. The data on glaciers were from the International Geosphere-Biosphere Programme, Data and Information Systems(IGBP-DIS),The IGBP DISCover global land cover product^46^, a global 1 km dataset, and Second Glacier Inventory Dataset of China^47^. For vegetation functional types, this study made use of Bonan’s^48^ inversion of relevant satellite data. The data provided by Bonan were also used to determine the stem area index and canopy height.

Terrestrial surface parameters are affected by human activities and climate change under non uniform conditions, leading to substantial changes of soil covers and vegetation functional types. This study aimed to reduce uncertainties of input data in such situations; this was achieved by improving the resolution of the input data. Another issue was that the driving field in this study has a resolution of 1 km, whereas CLM3.5 performs columnar assignments by grid. This means that data for the underlying surface must be configured at the same resolution and precision as that of the atmospheric forcing datasets; in other words, the number of grids within the study area has to be standardised to reduce the number of assignment errors and minimise the occurrence of disequilibria during simulations of various energy fluxes.

Following the aforementioned requirements, all raw terrestrial surface parameters with various coarse resolutions were refined to 1 km resolution. The following data were obtained from the Land–Atmosphere Interaction Research Group, Beijing Normal University (http://globalchange.bnu.edu.cn/): (i) soil texture: sand and clay contents (unit: %)^49^; and (ii) soil colour: 1 km resolution. The vertical changes of the soil properties (including sand and clay contents) down to 2.3 m were divided into eight layers: 0–0.045, 0.045–0.091, 0.091–0.166, 0.166–0.289, 0.289–0.493, 0.493–0.829, 0.829–1.383, and 1.383–2.296 m. On the other hand, the soil parameters (sand and clay) of the CLM surfdata were divided into ten layers for a vertical depth of 2.9 m: 0–0.007, 0.007–0.0279, 0.0279–0.0623, 0.0623–0.1188, 0.1188–0.212, 0.212–0.366, 0.366–0.619, 0.619–1.038, 1.038–1.727, and 1,727–2.864 m. Therefore, the data layers had to be matched via stratified interpolation and substitution.

Given the space constraints, only the substitution results for the soil texture parameters are shown. For the top six layers, pre- and post-substitution clay and sand parameters can be seen in Supplementary Fig. S1 online and Supplementary Fig. S2 online, respectively (from the top downwards: surfdata layers 1–6). The diagrams in the left and right panels are the raw data provided by CLM3.5 and new post-substitution parameters, respectively. The comparison of the two columns shows that the distribution of the raw data in the study area was very coarse and did not reflect the actual distribution of the soil texture. After interpolation and substitution, the data better reflected the actual soil texture conditions of multiple soil layers in Xinjiang. It was mentioned previously the glacier dataset was derived from the Second Glacier Inventory Dataset of China^47^. Resampling to 1 km resolution was performed before the data were added to the surfdata (the parameter module of CLM). The datasets for the other parameters (glaciers, lakes, and wetlands) were similarly re-sampled.

### Validation of SML data for Xinjiang

The data from SML stations were obtained from automatic soil moisture stations of the National Meteorological Information Center (NMIC) of the China Meteorological Administration (CMA) (Fig. 1). To evaluate results obtained during a later stage of this study, specific data were extracted from 54 c stations in Xinjiang; 29 are manual stations and the remaining are automatic stations. These data on the volumetric moisture content (unit: mm^3^/mm^3^) were obtained on an hourly basis and for seven soil layers: 0–10, 20–30, 30–40, 50–60, 70–80, and 90–100 cm. The volumetric moisture content of three soil layers was selected for this study: 0–10, 0–20, and 0–50 cm.

### Validation and analysis of SML simulation results

This study aimed to investigate the SML component in detail because SML changes can indirectly reflect a region’s hydrological and climatic conditions and SMLs are regularly used for numerical meteorological forecasts, prediction of mountain torrents, and monitoring of droughts that affect agricultural soils. Hourly data for ten soil layers in Xinjiang were acquired for SML modelling. Previously, ten spinning-up simulations to a depth of 80 cm were carried out for SMLs in Xinjiang. These provided the final equilibrium state for this component after it underwent all physical processes on the terrestrial surface. Hourly data on the volumetric moisture content of the soil were available for three layers only: 10, 20, and 50 cm. These data were obtained from 54 observation stations (SML Manual in Fig. 1) for the year 2012. As such, the hourly results generated by the CLM3.5 had to be subjected to weighted arithmetic processing using the data observed for the three soil layers. The volumetric moisture content for the three soil layers (0–10, 0–20, and 0–50 cm) of the CLM was eventually generated. The data were used for matching and model validation at a later stage.

### Describes the calculation method of taylor diagrams

Model data and observation data will be evaluated using Taylor diagrams^50^ in this research. The evaluation and analysis process was to eliminate any errors or biases arising from unaudited data recorded at the automatic stations. Among a total of 54 stations in the region, 16 moisture observation stations with higher-quality records were screened and selected using the quality control method and the corresponding simulation results were identified. The normalised standard deviation (*NSDV*) was then determined for the SML data of all three soil layers. For each layer, a CLM3.5-simulated value for each station (*S*_*i*_) and corresponding observed value (*G*_*i*_) were obtained. The factor for the standard deviations of those two values was then derived: *NSDV* = σ_*Si*_ / σ_*Gi*_. In addition, the simulated and observed values are also related to the correlation coefficient, amplitude margin (*E*), and bias. These relationships are shown in Eqs. (4) and (5)^50^:

4 $${E}^{2}=(RMS{E}^{2}-Bia{s}^{2})/{\sigma }_{{G}_{i}}^{2}$$

5 $${E}^{2}=NSD{V}^{2}+1-2\cdot NSDV\cdot R$$

Subsequent to calculations based on these equations, the Taylor diagram was used to plot the three evaluation indicators (*RMSE*, *NSDV* and *E*). The analysis showed that the correlation coefficient between *S*_*i*_ and *G*_*i*_ can be expressed using the angles on the diagram’s polar coordinates, while *NSDV* was expressed via the radial margin. The values from the observation stations (AWS) were plotted on the x-axis, where the values of both *R* and *NSDV* were 1. The amplitude *E* represents the distance between the AWS data and model data (which were pending evaluation). The smaller the value of *E* is, the higher is the accuracy of the model simulations.

## Results

### Spatial validation of the XJLDAS dataset and historical extremes

**Figure 2:**
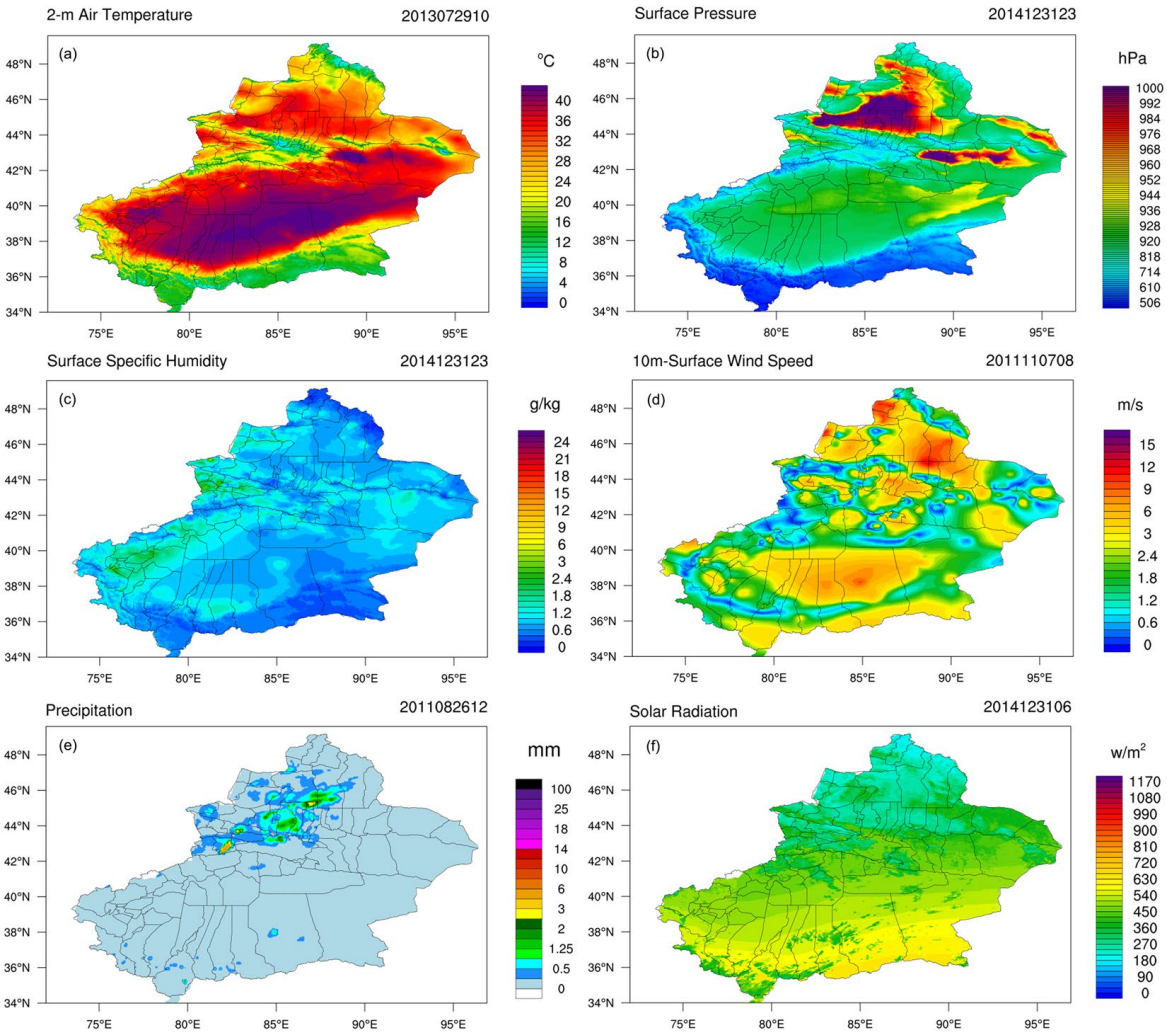
Spatiotemporal distributions of climate elements in Xinjiang based on the XJLDAS. The map was generated with NCAR Command Language (Version 6.6.2) [Software]. (2019). Boulder, Colorado: UCAR/NCAR/CISL/TDD. http://dx.doi.org/10.5065/D6WD3XH5.

The use of the LAPS/STMAS assimilation technique allowed to eventually build a 1 km atmospheric forcing datasets for Xinjiang for 2008–2013 (Fig. 2a–f). The historical data for the region indicated that extreme temperatures, precipitation, and wind velocities had severely affected the safety of humans and properties and led to destruction.

Several extreme meteorological disasters that had occurred in Xinjiang over the years and the timing and corresponding spatiotemporal distribution are given below:

- From July 29–31, 2013, historical maximum temperatures of 39.6 °C and 38.6 °C were reached in the Akto (Aketao) and Kargilik (Yecheng) counties, respectively.
- During the same time period, the maximum temperatures in Qira (Chira) County, Lop (Luopu) County, and Hotan (Hetian) exceeded 40 °C, reaching 42.1 °C, 41.9 °C, and 41.4 °C, respectively (Fig. 2a).
- On 7 November 7, 2011, the velocity of the sou’easter hitting Urumqi reached a historic extreme (Fig. 2d).
- On August 26, 2011, Urumqi experienced an extreme moment during a rare heavy rainstorm (Fig. 2e).

Spatial validation and analysis were performed for the aforementioned historical extremes and the corresponding XJLDAS dataset. The preliminary result for Xinjiang was that the XJLDAS-driven dataset could realistically reflect the hourly patterns of spatiotemporal changes of various meteorological parameters at the terrestrial surface.

### Applicability of the XJLDAS dataset to Xinjiang

The next step was to quantitatively analyse the accuracy of the XJLDAS-driven field for the CLM. The various evaluation indicators used for quantitative analyses of the 1 km forced field for Xinjiang, built in this study using LAPS/STMAS, included bias (BIAS), correlation coefficient (CORR), and root-mean-square error (RMSE). The intra-day accuracy was verified through matching the driven data to observed daily data (recorded at 0 am, 6 am, 12 pm, and 6 pm) obtained from 105 national automatic stations. The driving data were first subject to multi-year averaging (2009–2013). Next, bilinear interpolation was used to extract the driving field based on the latitudes and longitudes of the aforementioned 105 stations. Eventually, the matching multi-year daily averages (at the four times stated above) for the driving field were obtained for each station. (Due to space constraints, this paper only demonstrates the validation of the bias indicator for the entire Xinjiang Region.)

**Figure 3:**
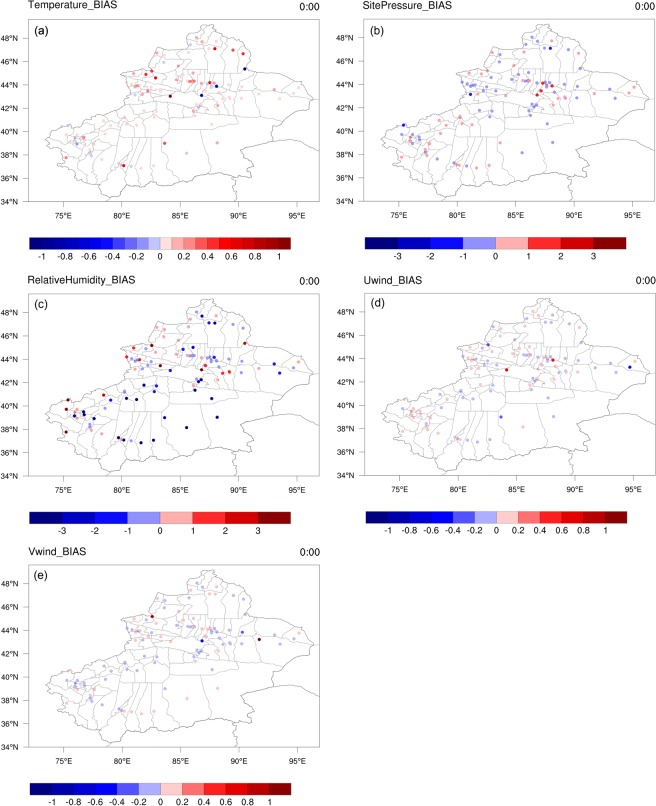
Intra-day (0 am) temperature, pressure, relative humidity, wind field biases in Xinjiang. The map was generated with NCAR Command Language (Version 6.6.2) [Software]. (2019). Boulder, Colorado: UCAR/NCAR/CISL/TDD. http://dx.doi.org/10.5065/D6WD3XH5.

The analytical results for the multi-year, intra-day (0 am–6 pm) bias indicators for temperature, pressure, humidity, and wind are shown in Fig. 3, Supplementary Fig. S3(a–c) online, Supplementary Fig. S4(a–c) online, Supplementary Fig. S5(a–c) online and Supplementary Fig. S6(a–f) online, respectively, after application of the LAPS/STMAS assimilation algorithm. The assessment results of intra-day (0 am–6 pm) biases in the temperature are shown in Fig. 3a and Supplementary Fig. S3(a–c) online.

The analysis indicated that all intra-day biases in the terrestrial surface temperature data for Xinjiang were small. At 0 am, the temperature biases of the entire region ranged between −1 and 1 K. The biases of the majority of stations varied between −0.2 and 0.2 K. There are only some stations that are located in the Altay Prefecture showing larger positive biases (0.8 K > BIAS > 0.6 K). Other stations with positive biases were found in the Bortala Mongol Autonomous Prefecture, Hotan, and Bayingolin Mongol Autonomous Prefecture, although the magnitudes were small (0.6 K > BIAS > 0.4 K). Negative biases (−0.8 K > BIAS > −1 K) were identified at three validation points located within the Changji Hui Autonomous Prefecture and Urumqi Region.

At 6 am, the biases of atmospheric temperatures showed an overall declining trend. The biases recorded earlier at 0 am were reduced to −0.2 to 0.2 K. Only three stations located at the intersection of the Bortala, Bayingolin, and Ili Kazakh autonomous prefectures maintained the biases of the earlier time (1 K > BIAS > 0.4 K). Two stations in the Changji Hui Autonomous Prefecture maintained the negative biases from 0 am. At 12 pm, most of the temperature biases within the region were small and generally ranged between −0.2 and 0.2 K. Small numbers of positive biases (1 K > BIAS > 0.8 K) were recorded at the intersection of the Bortala–Bayingolin–Ili prefectures and in the central region of the Altay Prefecture. The biases of atmospheric temperatures in all situations at 6 pm was quite similar to that at 12 pm.

The results of the assessment of intra-day (0 am–6 pm) biases in terrestrial surface pressure data are shown in Fig. 3b and Supplementary Fig. S4(a–c) online.

The biases were generally small in the whole region. At 0 am, the majority of the biases ranged from −1–1 Hpa. Detailed analysis showed that stations with larger positive biases (2 Hpa > BIAS > 1 Hpa) were mainly distributed in Urumqi. The Kizilsu Kyrgyz Autonomous Prefecture and Altay Prefecture each included one station with negative biases (− 1 Hpa > BIAS > −2 Hpa). Overall, the biases at 6 am were consistent with that at the earlier time, with Urumqi stations maintaining their positive biases (2 Hpa > BIAS > 1 Hpa). The Ili, Kizilsu, and Altay prefectures each had one station with negative biases (−1 Hpa > BIAS > −2 Hpa), while the biases of the remaining stations in Xinjiang generally varied from −1 to 1 Hpa. The biases at 12 and 6 pm ranging between −1 and 1 Hpa were similar to that at 6 am. In summary, based on the biases, the terrestrial surface pressure data established in this study were excellent.

Figure 3c and Supplementary Fig. S5(a–c) online show the assessment results of intra-day (0 am–6 pm) biases for the relative humidity. The performance of all XJLDAS-driven data for this parameter was satisfactory.

At 0 am, the overall biases for the region ranged between −3% to 3%; that of nearly half of the stations varied from −0.1% to 0.1%. Stations with larger positive biases (3% > BIAS > 1%) were located in the Ili, Bortala, and Kizilsu prefectures and Turpan Region. A wider range of minor negative biases (−1% > BIAS > −3%) was noted for other areas.

The biases of the majority of areas were reduced at 6 am. At this time, half of the region’s positive and negative biases initially recorded at 0 am declined to −2% to 1%. Relatively larger negative biases (−2% > BIAS > −3%) were recorded only in the Tian Mountains, which are located at the intersection of Shihezi, Changji Prefecture, and Urumqi Station. Larger positive biases (3%) were noted at four stations: two in the Kizilsu Prefecture, one in Kashgar, and one in the Bortala Prefecture. The biases of the remaining stations in the region ranged between −1% and 1%.

At 12 pm, the biases for the relative humidity varied from −3 to 2%, suggesting a good model performance. In fact, the biases maintained at −3 to 0% for most of the areas, with exceptions being the Kizilsu and Ili prefectures, where a small number of positive biases (1–2%) were registered. At 6 pm, most of the stations within the region showed biases between −1% and 1%.

The intra-day (0 am–6 pm) biases for zonal wind U and meridional wind V are shown in Fig. 3(d,e) and Supplementary Fig. S6(a–f) online, and it suggested CLM drivien by XJLDAS performed satisfactorily. For the U and V winds, the overall biases at 0 am were −0.4–0.4 m/s and −0.2–1 m/s, respectively. The biases in the combined wind parameter were generally between −0.2 and 0.2 m/s. Therefore, the CLM driven by XJLDAS performed satisfactorily for Xinjiang.

Detailed analysis indicated that stations with larger biases in the U wind were mainly distributed at two junctions: between the Changji Prefecture and Urumqi (one station) and between the Ili and Bortala prefectures (one station). All biases at these two stations were 0.6–0.8 m/s. Negative biases for this wind (−0.4 m/s > BIAS > −0.6 m/s) appeared at Alashankou in the Bortala Prefecture.

Larger V wind biases were identified for stations located in Alashankou, Hami, and Urumqi (one station per location). Specifically, minor positive biases (0.8 m/s > BIAS > 1 m/s) were found at the first two locations, while negative biases (−0.4 m/s > BIAS > −0.6 m/s) were registered at the third location. The U and V wind biases at the Alashankou Station were completely opposite.

The overall biases for the U and V winds at 6 am were −0.6–0.8 m/s, with most of the stations having biases close to that at 0 am. This indicated stability. For the U wind, negative biases (−0.4 m/s > BIAS > −0.6 m/s) were observed at the Alashankou Station, while positive biases (0.2 m/s > BIAS > 0.6 m/s) were recorded at the Wenquan Station. Urumqi and the Altay Prefecture each included one station with negative biases (−0.4 m/s > BIAS > −0.8 m/s). For the V wind, biases for most stations in Xinjiang at 6 am ranged between −0.4 m/s and −0.6 m/s. Four stations in the Turpan Area exhibited definite negative biases (−0.2 m/s to −0.6 m/s), while positive biases at the Alashankou Station reached 1 m/s.

At 12 pm, the biases of the U and V winds for the entire region were −0.6–0.8 m/s, that is, close to that at 6 am. For the most stations, the biases were limited to −0.2–0.2 m/s. The U wind at the Alashankou Station had negative biases similar to that at 6 am (−0.4 m/s > BIAS > −0.6 m/s). The same is true for the Wenquan Station but with respect to positive biases (0.2 m/s > BIAS > 0.6 m/s). There were also minor negative biases (−0.4 m/s) in Urumqi and the Bortala Prefecture. The V wind biases at 12 pm were within the range of −0.2 m/s and 0.2 m/s. The same four stations in the Turpan Area continued to exhibit definite negative biases (−0.2 m/s to −0.4 m/s). At the Alashankou Station, positive biases reached 1 m/s, similar to the situation at 6 am.

The regional biases for the two winds were between −0.8 m/s and 0.8 m/s at 6 pm, with that for the most stations being −0.2–0.2 m/s. The U wind had negative biases (−0.4 m/s > BIAS > −0.6 m/s) at the Alashankou Station and minor negative biases (−0.4 m/s to −0.6 m/s) in Hami and at the Turpan Station. For most of the region’s stations, the V wind biases were between −0.2 m/s and −0.2 m/s. Minor negative biases (−0.2 m/s to −0.6 m/s) were observed in Hami and at the Turpan and Urumqi stations. The positive biases at the Alashankou Station reached 0.8 m/s.

In general, it can be concluded that the XJLDAS driving field was able to accurately reproduce the spatial distribution of various terrestrial surface parameters in Xinjiang.

### Analysis of intra-annual spatiotemporal changes of SML in Xinjiang

**Figure 4:**
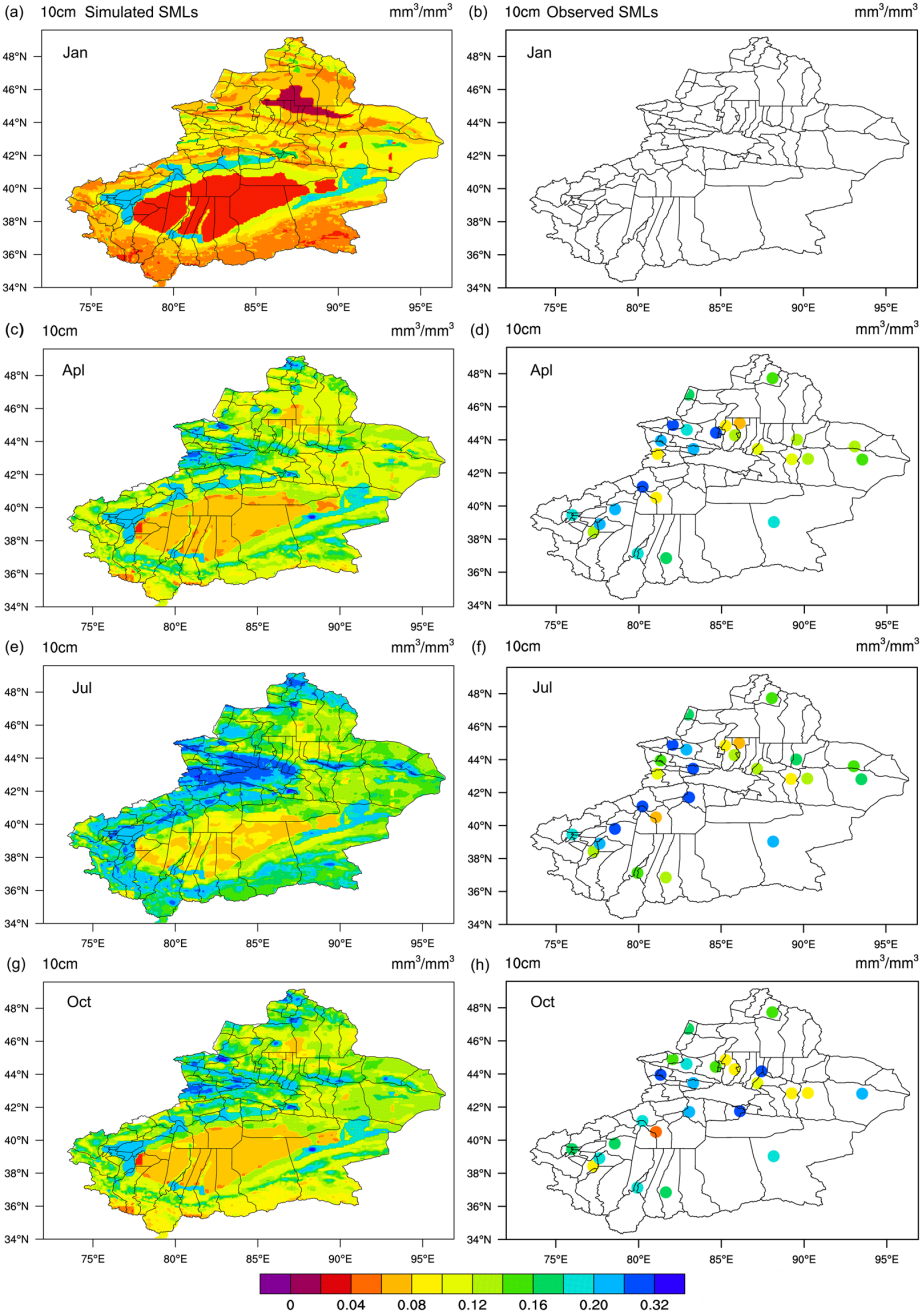
Monthly trends for simulated versus observed SMLs of the 0–10 cm soil layer in 2012 (unit: mm^3^/mm^3^). The map was generated with NCAR Command Language (Version 6.6.2) [Software]. (2019). Boulder, Colorado: UCAR/NCAR/CISL/TDD, http://dx.doi.org/10.5065/D6WD3XH5.

The hourly SML results for Xinjiang in 2012 generated by XJLDAS and CLM3.5 were used to analyze and better understand the patterns of intra-annual changes in the region’s SML distribution from a spatiotemporal perspective. The data were extracted according to the three soil layers (0–10, 0–20, and 0–50 cm) and the monthly mean values were calculated. The corresponding observed data for the three soil layers were then obtained from 29 manual observation stations (alternate red–yellow dots in Fig. 1), which provided more accurate data for the region. These were used to validate the model outputs. Among the three layers, the SML changes for the 0–10 cm layer had a larger magnitude. Hence, details are only provided for this soil layer. The changing trends in the SMLs of the terrestrial surface (0–10 cm) in 2012 generated by the XJLDAS dataset-driven CLM3.5 are shown in Fig. 4 (left panel).

The analysis of Fig. 4(a–h), Supplementary Fig. S7(a–h) online and Supplementary Fig. S8(a–h) online revealed that there was an excellent consistency between the SMLs simulated by the XJLDAS and CLM3.5 and data observed at the corresponding stations. The overall intra-annual trend for the region was a gradual moisture increase over time. This was maintained until May–August; the maximum values were reached in June–July. Towards the end of the year, the trend changed to a gradual decline (the observed value was missing because SMLs at 0–10 cm were not recorded in Xinjiang).

A detailed and monthly analysis of the SMLs in the 0–10 cm layer of Xinjiang was carried out next. The first occurrence of large-scale moisture increase occurred between March and April each year. In most areas within Xinjiang, the SMLs rapidly increased from 0.04–0.08 mm^3^/mm^3^ to 0.08–0.12 mm^3^/mm^3^ in February. This trend was particularly evident in the Tarim and Junggar basins, where the SMLs rose quickly from 0–0.06 mm^3^/mm^3^ in February to 0.04–0.12 mm^3^/mm^3^ in March. Within the entire region, SML changes were especially apparent from March to April, with significant increases in the Altai Mountains, at the northern and southern slopes of West Tian Mountains, and in the East and West Kunlun Mountains. Over two months, the SMLs at the Altai Mountains increased from 0.12–0.14 mm^3^/mm^3^ to 0.19–0.25 mm^3^/mm^3^, while that of the northern and southern slopes of West Tian Mountains quickly increased from 0.08–0.18 mm^3^/mm^3^ to 0.14–0.32 mm^3^/mm^3^. The southern slope of East Tian Mountains also experienced a sudden change and large-scale increase in soil moisture. The SMLs of large areas in the East and West Kunlun Mountains increased from 0.04–0.18 mm^3^/mm^3^ to 0.06–0.25 mm^3^/mm^3^.

We also found that the rapid rise in the region’s SMLs was caused by snow (ice) melting in spring (snowmelt in Xinjiang occurs during March–April every year). The trends in Supplementary Fig. S7(c) online and Supplementary Fig. 4(c) online show that areas with significant SML increases are located at or near mountainous areas with thick snow cover. The region’s weather becomes hot from May until the end of September. Precipitation occurs more often during this season, causing alpine ice and snow to melt. This in turn causes significant SML fluctuations and increases in the region. Fig. S7(e–g), Figs. 4(e) and S8(a) show that this trend is evident at the northern and southern slopes of the East and West Tian Mountains, Ili River Valley, Altai Mountains, and peripheries of the Kunlun Mountains. During this period (May–September), the northern and southern slopes of the East Tian Mountains show more concentrated SML distributions with greater magnitudes of change. The peak values (>0.32 mm) for the region occur in July, with the magnitude of increase being the greatest in a year, and this was mainly caused by snow melting and precipitation in July.

October is the last month of a year with high SMLs; an overall downward trend can be observed thereafter. The sudden decline in SMLs during the winter season is likely caused by the steep drop in atmospheric temperatures, which causes the soil moisture to freeze. This hypothesis will be further validated in a later section of this paper. After comparative analysis between the SML results simulated by XJLDAS and CLM3.5 and observations, it was concluded that the model could very accurately reproduce the spatiotemporal changes of SMLs in Xinjiang.

### Validation of the time series for the SML changes in Xinjiang

To evaluate the model performance in SML simulation, we used measured SMLs at 54 stations in Xinjiang at three soil depths (Fig. 1).

The CLM3.5 provided SML outputs for ten soil layers: 0.007, 0.0279, 0.0623, 0.1188, 0.212, 0.366, 0.619, 1.038, 1.727, and 2.846 m. For the purpose of regional SML validation, a weighting coefficient (soil depth) was used to match the values simulated by the model (extracted based on the three different soil layers) to the values recorded at the 54 soil moisture observation stations for the corresponding layers.

**Figure 5:**
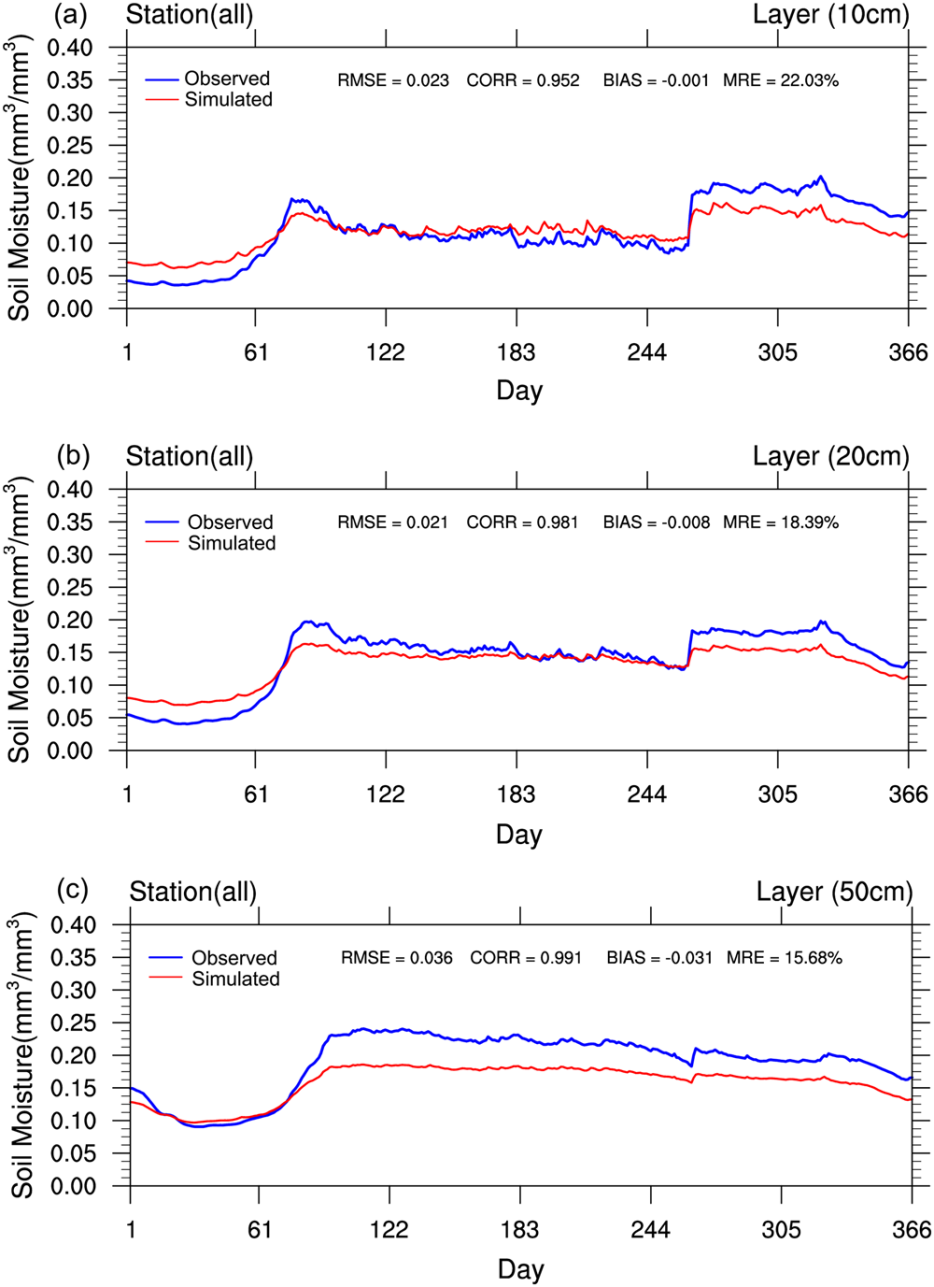
Time series ofthe SMLs forthree soil layers (10, 20, and 50 cm) at 54 stations in Xinjiang in 2012.

First, the various time series reflecting SML changes for Xinjiang in 2012, as simulated by XJLDAS and CLM3.5, were derived. The respective time series based on the daily distribution after averaging the observed values of the 54 stations for the three different soil layers are shown in Fig. 5(a–c), representing the layers at 0–10, 0–20, and 0–50 cm, respectively. In addition to the trends of simulated and observed values, Fig. 5(a–c) also present the RMSE, CORR, BIAS, and mean radial error (MRE) for the three layers.

The overall CORR of the simulations for each soil layer was greater than 0.952. The simulation results of XJLDAS and CLM3.5 were good, relative to the observed results for the three soil layers. The RMSE of the 0–50 cm layer was worse than that of the other two layers. The BIAS of the 0–10 cm layer (−0.001) was slightly better than that of the 0–20 cm layer (−0.008). However, the former performed worse than the latter with respect to CORR (0.952), RMSE (0.023 mm^3^/mm^3^), and MRE (22.03%). This indicated that XJLDAS and CLM3.5 had the best simulation capabilities for the second layer (0–20 cm), followed by the first (0–10 cm) and third (0–50 cm) layers.

These results might have been affected by two factors: the parameterisation scheme selected for the model; and inherent issues of the observed data. The latter refers to SMLs having greater fluctuations because they were affected by variations of near-ground meteorological factors (such as precipitation, snowfall, solar radiation, and atmospheric temperature). With respect to automatic observation stations, there are inherent issues with signal sensitivities.

In terms of biases in SMLs, a greater underestimation between the simulated and observed results was observed for the third layer compared with the other two layers (for which the simulation results were only slightly underestimated). Between early March and April 2012, the overall trend of SMLs recorded at the 54 stations was a substantial increase. The lowest levels were recorded in January–February, which is winter in the region with seasonal frozen soils prevailing. When temperatures rise, the snow and frozen soil rapidly melt in March–April, resulting in steep SML increases. A second major and sudden increase in SMLs occurs from the end of September to the beginning of October each year. The main reasons likely are alpine snowmelt and precipitation during July–September.

After analysing the simulated and observed SMLs for the three soil layers, the maximum values of the former were found to be smaller than that of the latter for early March and September every year, indicating that the model’s detection capability of SMLs needs to be fine-tuned for these two periods. Further analysis revealed that these two periods coincided with high incidences of snowmelt and precipitation within the region, respectively. To minimise the bias in the maximum values, the author aims to improve the parameterisation scheme of related physical processes at a later stage.

From the perspective of soil layers, the SML increases in all three layers exhibited an overall upward trend with depth. From the seasonal perspective, SMLs in Xinjiang were generally higher in summer compared with winter.

### Taylor diagram analysis of SMLs in Xinjiang

In this section, the Taylor diagram was used to analyse the differences between the correlation coefficient and standard deviation of SMLs recorded at the soil moisture observation stations in Xinjiang. The aim was to conduct a fast and convenient evaluation of the model simulation results using various statistical indicators and examine the fitting results for the different soil layers and various observation stations.

**Figure 6:**
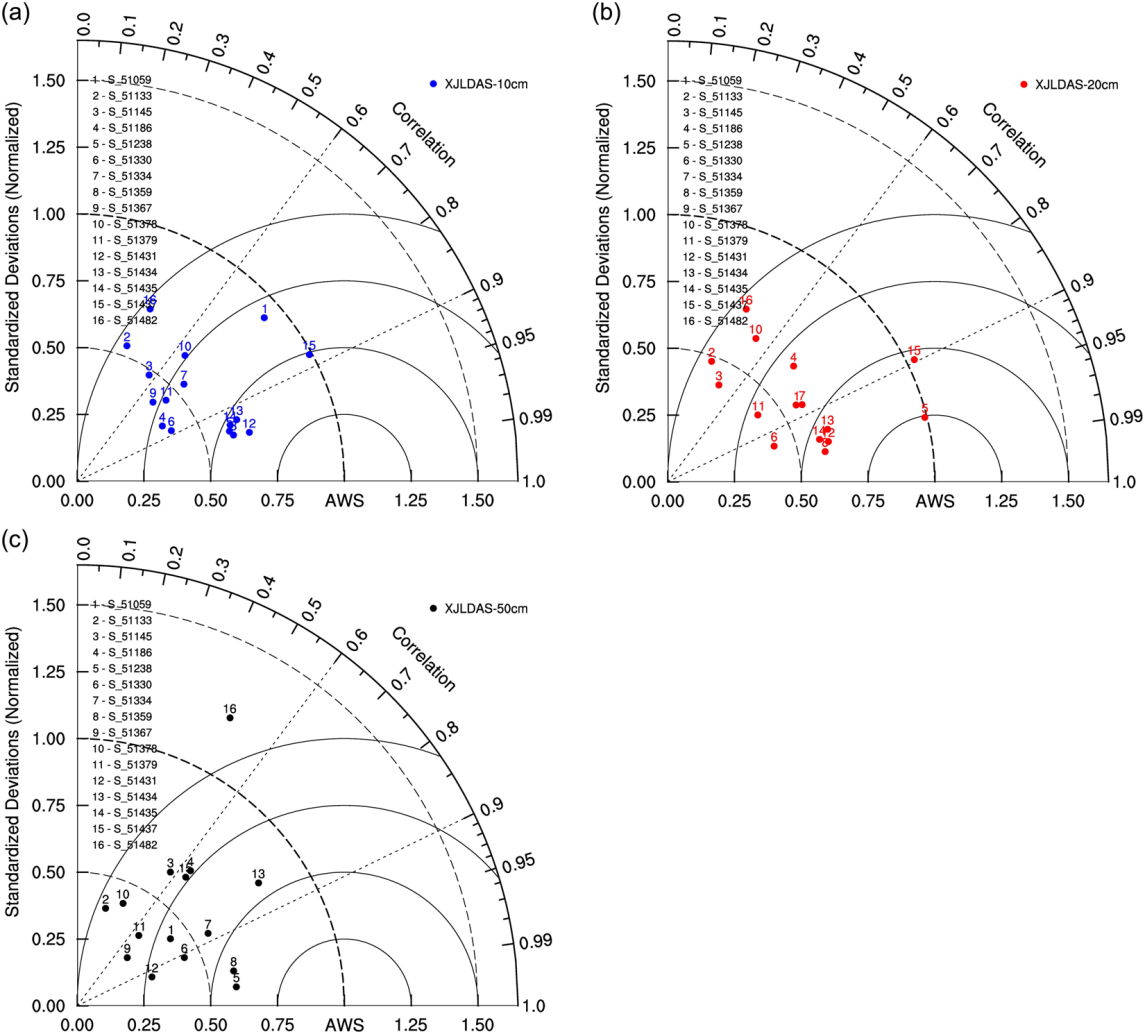
Taylor diagram for SMLs of the 0–10 cm, 0–20 cm and 0–50 cm layers for 16 stations in Xinjiang in 2012.

For the 16 national SML stations in Xinjiang, the Taylor diagrams for the three soil layers (0–10, 0–20, and 0–50 cm) in 2012 are shown in Fig. 6(a–c), respectively. The numbers in the diagram refer to the SML stations (please see the legend for details on specific stations). In the diagram, AWS represents the observed values. Overall, the XJLDAS and CLM3.5 simulations of SMLs of the three layers were ideal. The correlation coefficients for most of the simulated results of the 0–10 cm layer were in the range of 0.6–0.96 (Fig. 6a). This proved that the overall performance capabilities of the proposed model (XJLDAS and CLM3.5) were superior to that of CLDAS when applied to Northwest China^51^.

Comprehensive analysis showed that the NSDs for all 16 stations were within 1 and that for Stations 3, 4, 6, 9, and 11 were within 0.5. The correlation coefficients for Stations 8, 11, 12, and 13 were the highest, while the simulation results for Station 15 were good.

The correlation coefficients of simulated SMLs of the 0–20 cm layer were mostly 0.7–0.98 (Fig. 6b), that is, better than that of the 0–10 cm layer. Nevertheless, those for Stations 2, 3, and 16 were still poor. Compared with other stations, the simulated results for the 0–20 cm layer at Station 5 were closer to the observed values. For this layer, with the exception of Stations 3 and 15, the NSDs for most of the stations were at approximately 0.5.

Figure 6c shows that the correlation coefficients for SMLs of the 0–50 cm layer were 0.6–0.99. Although the correlation coefficients were excellent, the simulated values were far from the observed values, except for Stations 5 and 8. Among the 16 stations, the NSDs for almost half of the stations were within 0.5, while that for the remaining stations were distributed around 0.6 (except for Station 16). The simulation results for all three layers at Station 16 were poor, especially in case of the third soil layer.

## Discussion

The water of the Ebinur Lake has gradually become more mineralised over the last few years. The conflict between the watershed ecology and water for domestic use has also become more apparent. In addition, the land has seriously degraded. 1,500 km^2^of lakes in this basin has degraded to salt deserts. The size of salinised areas reaches 71 km^2^.The variations in the soil moisture distribution within the basin are as vast as that in the climatic distribution, inducing corresponding responses of the vegetation cover.

The aim of this study was to validate the model performance in SML simulation and examine the spatiotemporal features and changes in SMLs within Xinjiang. Analyses and evaluations were made based on multiple viewpoints including time series and Taylor diagrams. The analysis and validation of simulated SML results focused on the Bortala–Jing River Basin and include time series validation and analyses of multivariate spatiotemporal responses. The findings indicated that the results simulated by the XJLDAS-driven CLM3.5 and data recorded at observation stations were generally consistent. The intra-annual analysis of SML changes within the region revealed that the main impact factors were precipitation and snowmelt. There was a trend of gradual SML increases as the year progressed, which was maintained until May–August. The Bortala–Jing River Basin, the focus of the study area, similarly showed a gradual increase in SMLs from April until the end of September.

Compared with the observed results for the three soil layers, the simulated SMLs were generally underestimated to a certain extent. The SML trends for the three soil layers at all 54 Xinjiang stations in 2012 showed larger increases from early March to April and from the end of September to early October. These phenomena were largely due to alpine snowmelt and increased precipitation. Based on the Taylor diagram that was used to analyse the correlation coefficients and standard deviations between the data recorded at all SML observation stations in the region and the simulated SMLs for all three soil layers by the XJLDAS-driven CLM3.5, the latter were quite accurate. In fact, for Northwest China, the performance capabilities of the proposed model were superior to that of the CLDAS products released by the CMA^51^.

There are some studies that used CLM for large-scale soil moisture evaluation; however, it is difficult for the coarse spatial and temporal resolution (1° and 3-hour time step) analysis to reflect detailed changes^23,52^. The comparison with those previous studies^51,53^ suggested that our study exhibited more detailed presentation and analyses due to improve both atmospheric forcing data and the land surface parameters. Furthermore, although the Jinghe River and Bohe River basins are quite vulnerable and representative in China, very few studies assessed soil moisture in this area. For example, Liu^54^ used remote sensing data for retrieving soil moisture. This study is a good attempt, and it also further analyzed the performance of the XJLDAS and CLM3.5 in the Bortala–Jinghe River Basin. Based on time series validation, the model worked well. The next step was to validate the hypothesis that snowmelt in the region was a major contributor to SML changes. An hourly response analysis of SMLs in the Bortala–Jing River Basin was performed relative to the snowmelt parameter, and it was found that snowmelt contributed to SMLs in March–April. In conclusion, the XJLDAS atmospheric forcing datasets was able to very effectively drive the CLM3.5 for 1 km high-resolution simulations of the terrestrial surface processes in Xinjiang. In future, the proposed forcings field can also enhance the high-resolution simulations of other land models in China.

### Validation and analysis of SML simulation results for the Bortala–Jing River Basin

**Figure 7:**
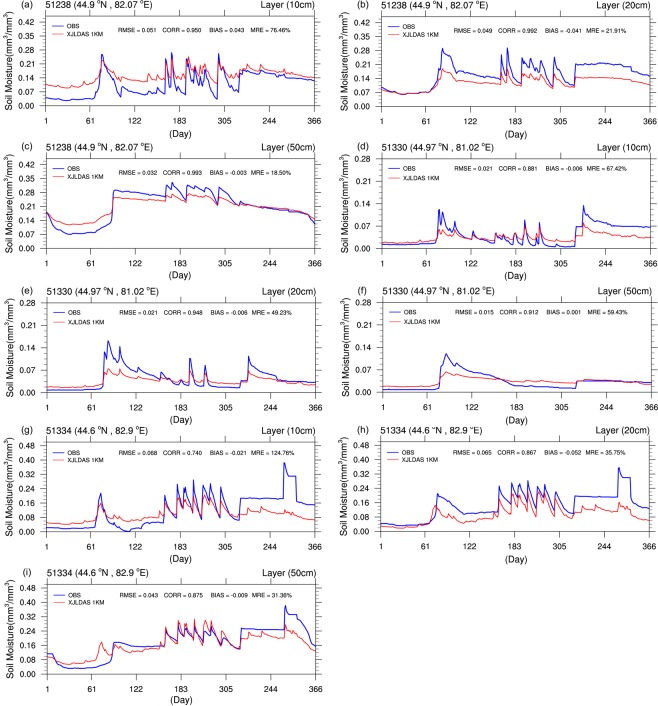
Time series of SMLs for the three soil layers (10, 20, and 50 cm) at each of the four stations in the Bortala–Jing River Basin in 2012.

The evaluation of the daily fitting results between the observed and simulated values for the three soil depths (10, 20, and 50 cm) at four stations in the Bortala–Jing River Basin for 2012 is shown in Fig. 7(a–i). The horizontal and vertical axes represent the simulation time (day) and SMLs (mm^3^/mm^3^), respectively. Based on the evaluation of the results of various models, the XJLDAS-driven CLM3.5 can accurately simulate the patterns of SML changes for the three different soil layers at the Bortala–Jing River Basin. For the first layer (10 cm) of Station 51334, the correlation coefficient of the model was lower (Fig. 7g); that for the 20 and 50 cm layers were comparatively better (Fig. 7). Because the simulation results for the Bortala–Jing River Basin were evaluated in the previous section using the Taylor diagram section, differences in the NSDs for the watershed will not be analysed here.

Based on the detailed analysis of Fig. 7, the SMLs in the basin exhibited the following phenomena within the year:

- The first sudden increase in SMLs occurred after the first 60 days of the year (i.e., March–April), which was probably due to snowmelt in the region during spring.
- After the first sharp rise, the SMLs in the Bortala–Jing River Basin were in a state of fluctuation after the first 150 days of the year (i.e., May–October). This state persisted until October. This is the period with high precipitation and fast temperatures increases of the terrestrial surface. We concluded that, on one hand, the fluctuations were due to precipitation change; on the other hand, evaporation led to the decline in SMLs.
- Another sudden rise in SMLs occurred in the end of October; the moisture levels remained constant until the end of the year. This was likely due to the cold October air producing precipitation (snow).
- Eventually, the SMLs within the study area maintained a stable and constant state for the rest of the year.

### Analysis of intra-annual spatiotemporal changes of SMLs in the Bortala–Jing Rivers Basin

The earlier analysis confirmed a large increase in SMLs in Xinjiang around March–April each year, which may be caused by snowmelt in the region during this period. After all, the Bortala–Jing River Basin in the study area is located near the northern and southern slopes of the West Tian Mountains and the Ili River Valley. To verify if a significant correlation exists between the region’s snowmelt period and its SMLs, the following variables were extracted for the basin in April 2012: (i) snowmelt at 02–12 UTC on April 9, a single day with more snowmelt; and (ii) the model’s hourly output of SMLs. The hourly snowmelt within the region is shown in Supplementary Fig. S9(a–m) online, while the corresponding spatial changes of the SMLs are shown in Supplementary Fig. S9(a1–m1) online.

The comparison between the two sets of diagrams revealed that areas with snowmelt were relatively wetter. There was no notable sign of snowmelt at 00–01 UTC, during which the corresponding SMLs tended to be stable and remained at 0.14–0.25 mm^3^/mm^3^. Areas with higher SMLs included the wetlands surrounding the Ebinur lake (0.25 mm^3^/mm^3^) and the glacier-covered areas of the Tian Mountains (0.32 mm^3^/mm^3^). At 02 UTC (10 am in Beijing), the watershed experienced more snowmelt. Random small-scale increases were recorded in the wetlands surrounding the Ebinur lake, ranging from 0.25 mm^3^/mm^3^ to more than 0.32 mm^3^/mm^3^.

Widespread snowmelt occurred at the intersection between the northern and southern slopes of the West Tian Mountains, which resulted in substantial SML increases near the southern slope at 02 UTC (Supplementary Fig. S9(C1) online) and in the Bortala–Jing River Basin. This trend was especially notable in the entire basin when the snowmelt intensified. The snowmelt reached a high level at 03 UTC (Supplementary Fig. S9(d) online), causing SMLs to increase by a large magnitude (0.25–0.32 mm^3^/mm^3^) around the Sayram Lake located within the watershed. At this time, the SMLs for most areas at the southern slope had also reached a daily high (0.25–0.32 mm^3^/mm^3^). With the gradual disappearance of the snowmelt, the widespread increases in SMLs also stopped at 12 UTC.

The analysis for the period from 03 to 12 UTC further revealed that, despite the cessation of snowmelt on a regional basis, the process continued at the Sayram Lake. This caused the areas around the lake to maintain high SMLs. It is this snowmelt supply that replenished the lake waters. Although the areas around the Ebinur lake also experienced SML increases during snowmelt, these changes were mainly concentrated in peripheral saline and alkaline soils. The snowmelt supply contributed more to SML increases in areas around the Sayram Lake than that around the Ebinur lake.

Based on the analysis of SMLs in the study area on April 9, 2012, there was a good match between the distribution of snowmelt and areas in the Bortala–Jing River Basin with higher SMLs. This indicates that the substantial SML increases in March–April were, to a large extent, triggered by snowmelt.

## Supplementary information

Supplemrntary Figure
