# Supplementary material for: High-resolution simulation and validation of soil moisture in the arid region of Northwest China
Source: Sci Rep. 2019 Nov 21;9:17227. doi: 10.1038/s41598-019-52923-x (PMC6872663; doi:10.1038/s41598-019-52923-x)
Supplement: Supplementary file 1 — Supplemrntary Figure [file 41598_2019_52923_MOESM1_ESM.pdf]

SUPPLEMENTARY FIGURES

High-resolution simulation and validation of soil moisture in the arid region of Northwest China

Xianyong Meng[1,2], Hao Wang[3], Ji Chen[2], Mingxiang Yang[3], Zhihua Pan[1]

[1] College of Resources and Environmental Science, China Agricultural University (CAU), Beijing 100094, China

[2] Department of Civil Engineering, The University of Hong Kong (HKU), Pokfulam 999077, Hong Kong, China

[3] State Key Laboratory of Simulation and Regulation of Water Cycle in River Basin & China Institute of Water Resources and Hydropower Research, Beijing 100038, China

SUPPLEMENTARY FIGURES

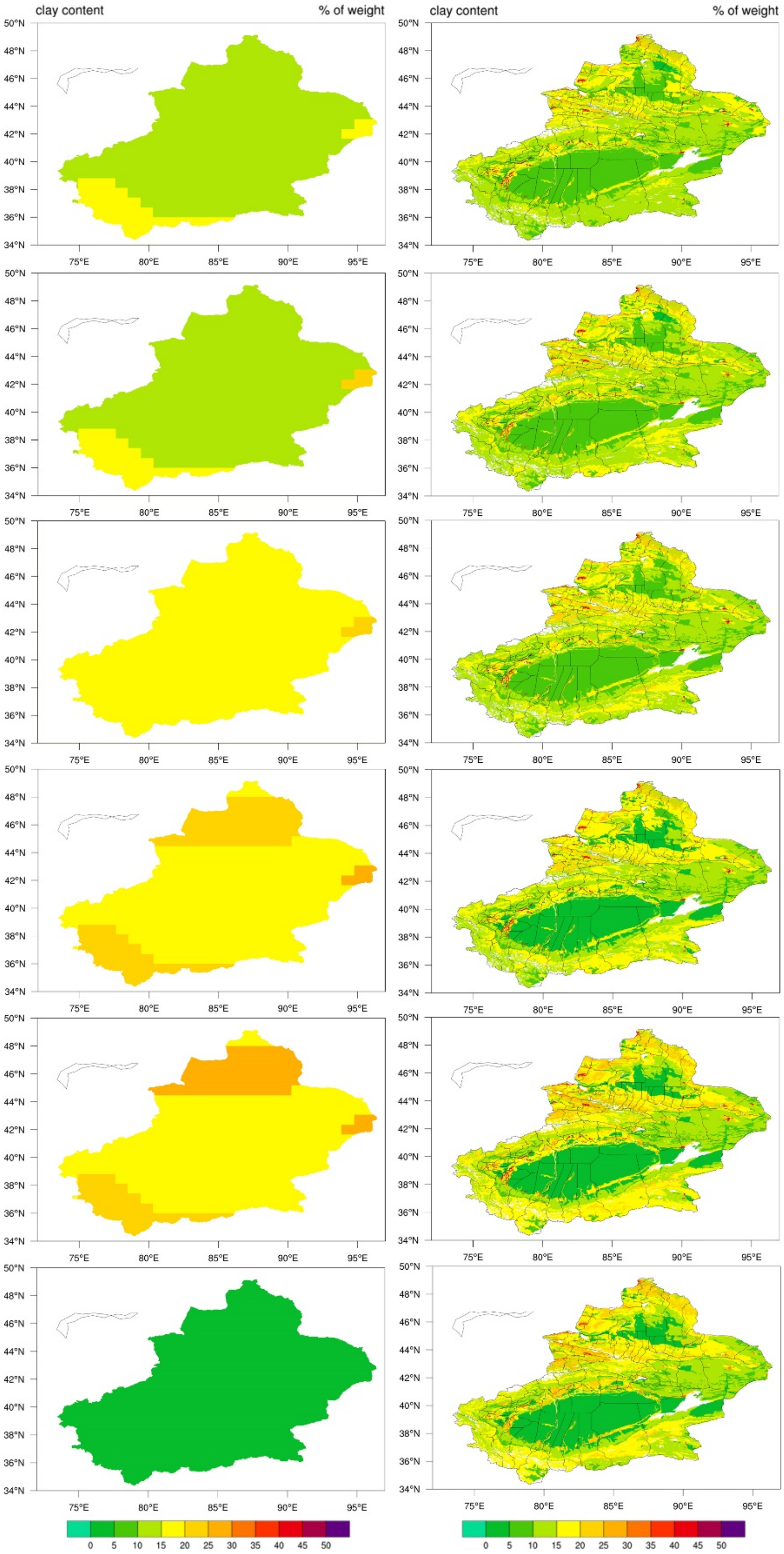

Supplementary Figure S1: Comparison of the clay parameter pre- and post-substitution (wt%). The map was generated with NCAR Command Language (Version 6.6.2) [Software]. (2019).Boulder,Colorado: UCAR/NCAR/CISL/TDD. http://dx.doi.org/10.5065/D6WD3XH5.

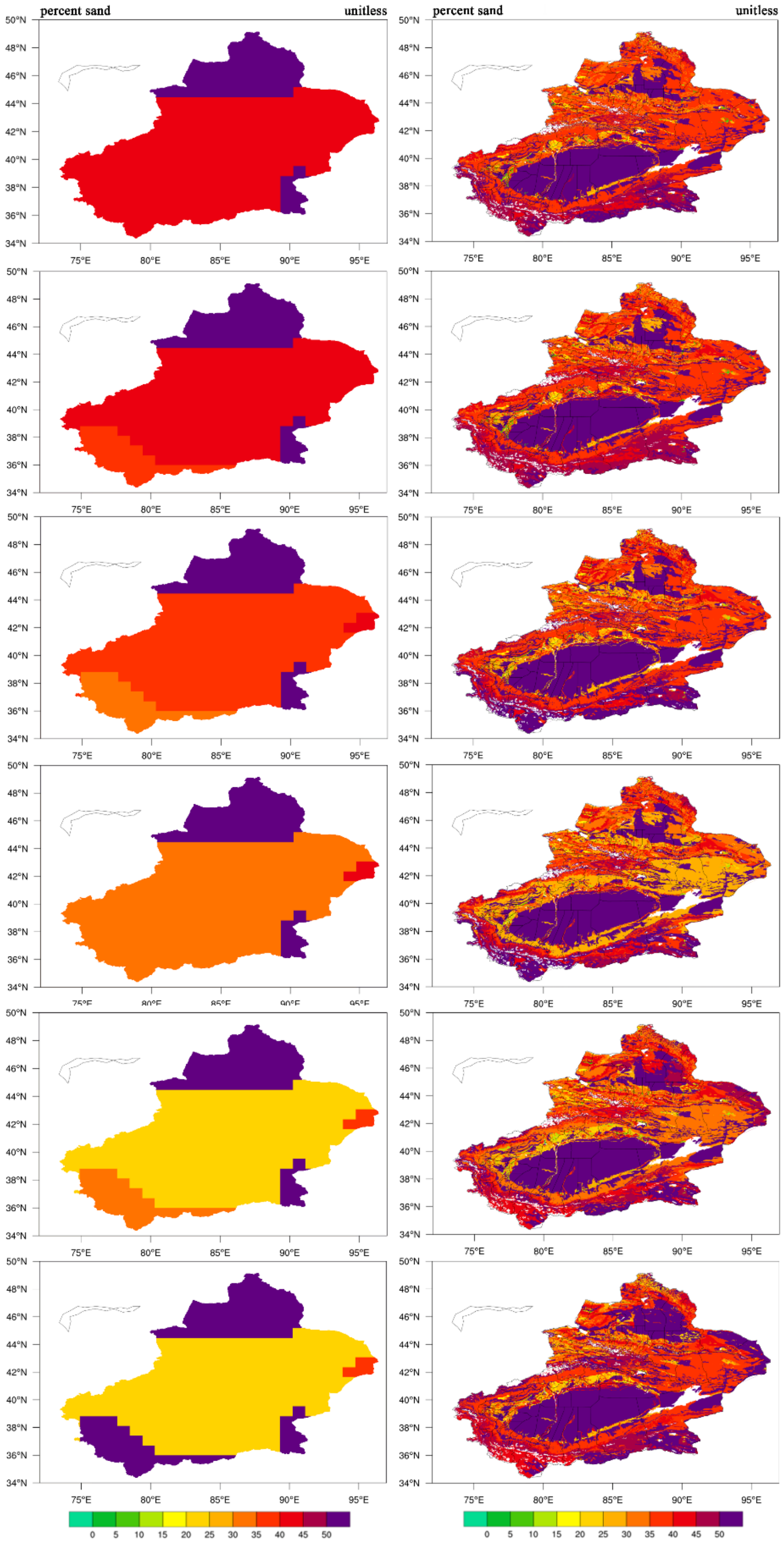

Supplementary Figure S2: Comparison of the sand parameter pre- and post-substitution (unitless). The map was generated with NCAR Command Language (Version 6.6.2) [Software].(2019).Boulder,Colorado:UCAR/NCAR/CISL/TDD.http://dx.doi.org/10.5065/D6WD3XH5.

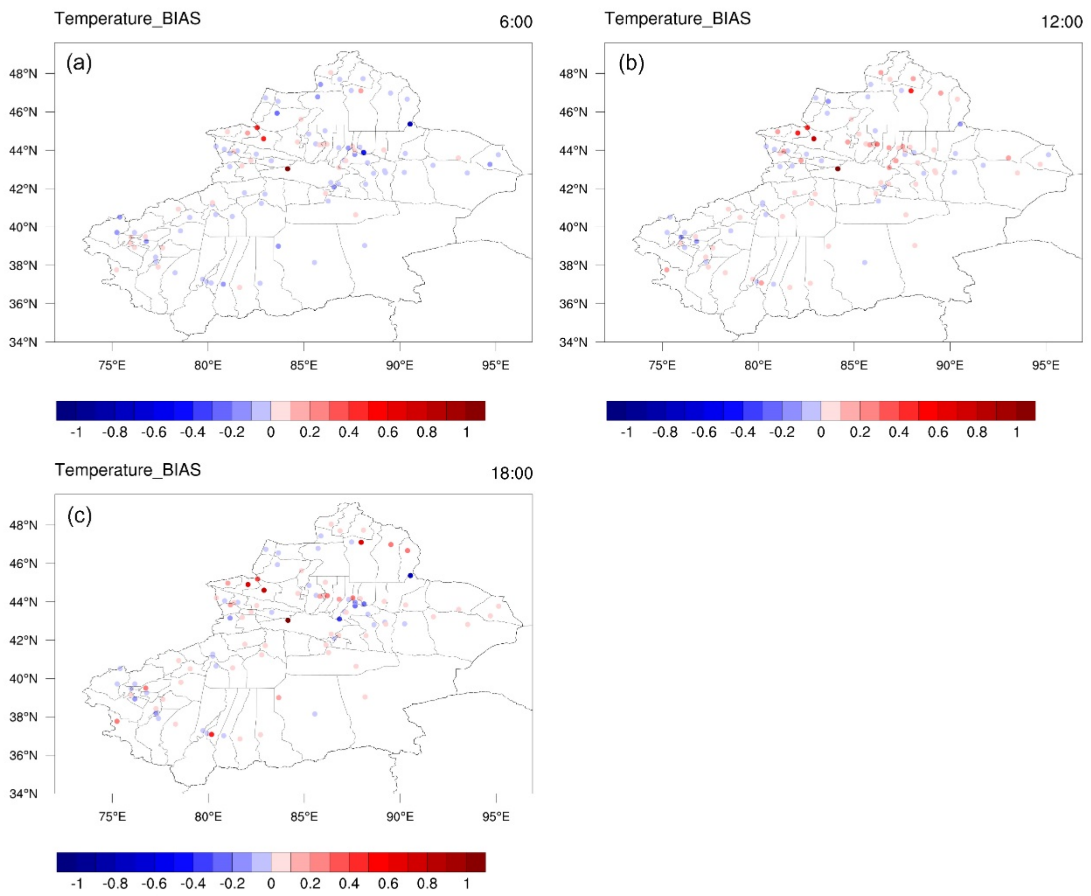

Supplementary Figure S3: Intra-day (6 am-6pm) temperature biases in Xinjiang. The map was generated with NCAR Command Language (Version 6.6.2) [Software]. (2019). Boulder, Colorado: UCAR/NCAR/CISL/TDD. http://dx.doi.org/10.5065/D6WD3XH5.

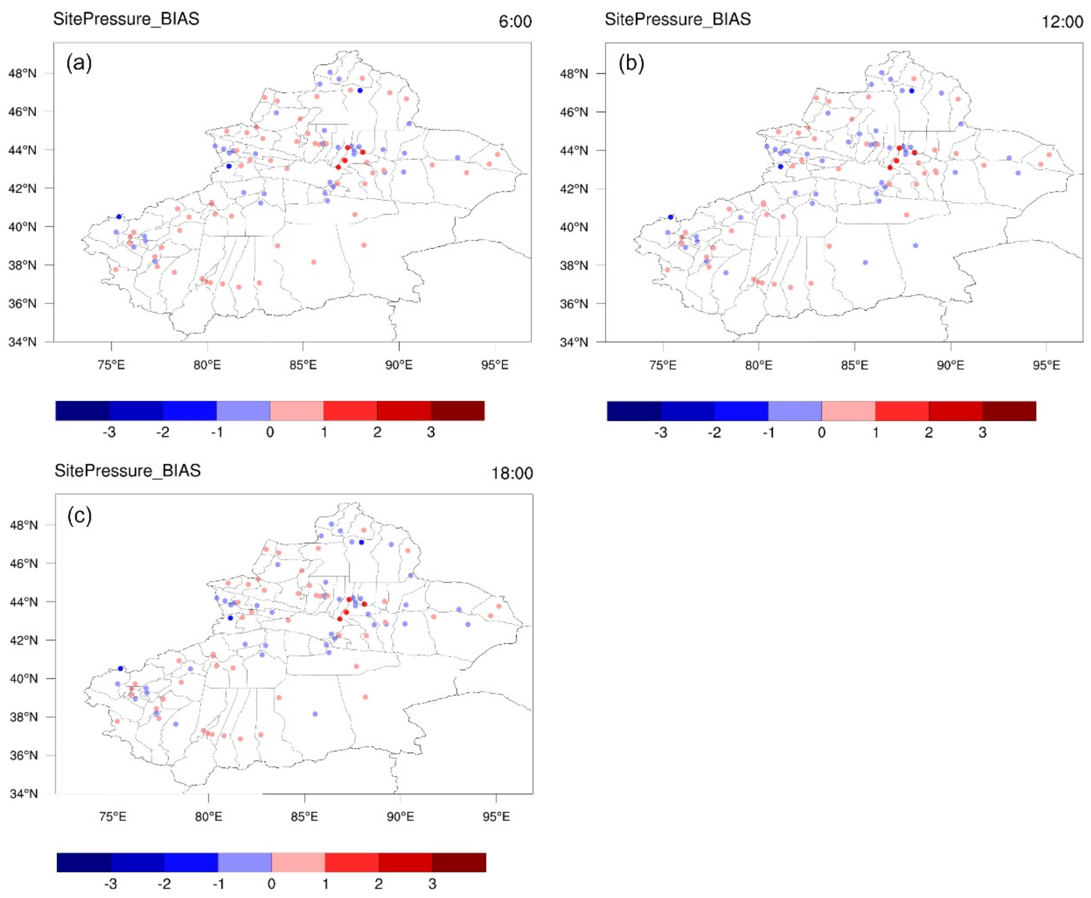

Supplementary Figure S4: Intra-day (6 am-6pm) pressure biases in Xinjiang. The map was generated with The map was generated with NCAR Command Language (Version 6.6.2) [Software].(2019).Boulder,Colorado:UCAR/NCAR/CISL/TDD. http://dx.doi.org/10.5065/D6WD3XH5.

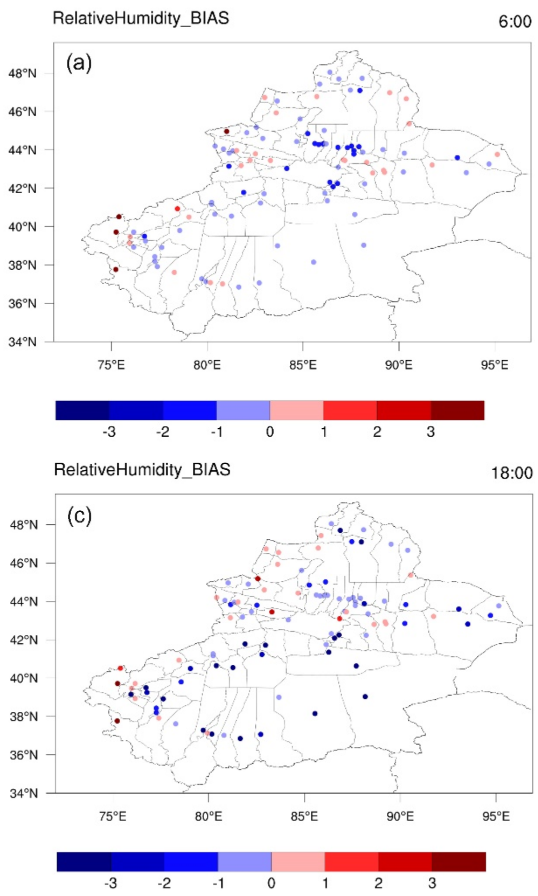

Supplementary Figure S5: Intra-day (6 am-6pm) relative humidity biases in Xinjiang. The map was generated with NCAR Command Language (Version 6.6.2) [Software]. (2019). Boulder, Colorado:UCAR/NCAR/CISL/TDD. http://dx.doi.org/10.5065/ D6WD3XH5.

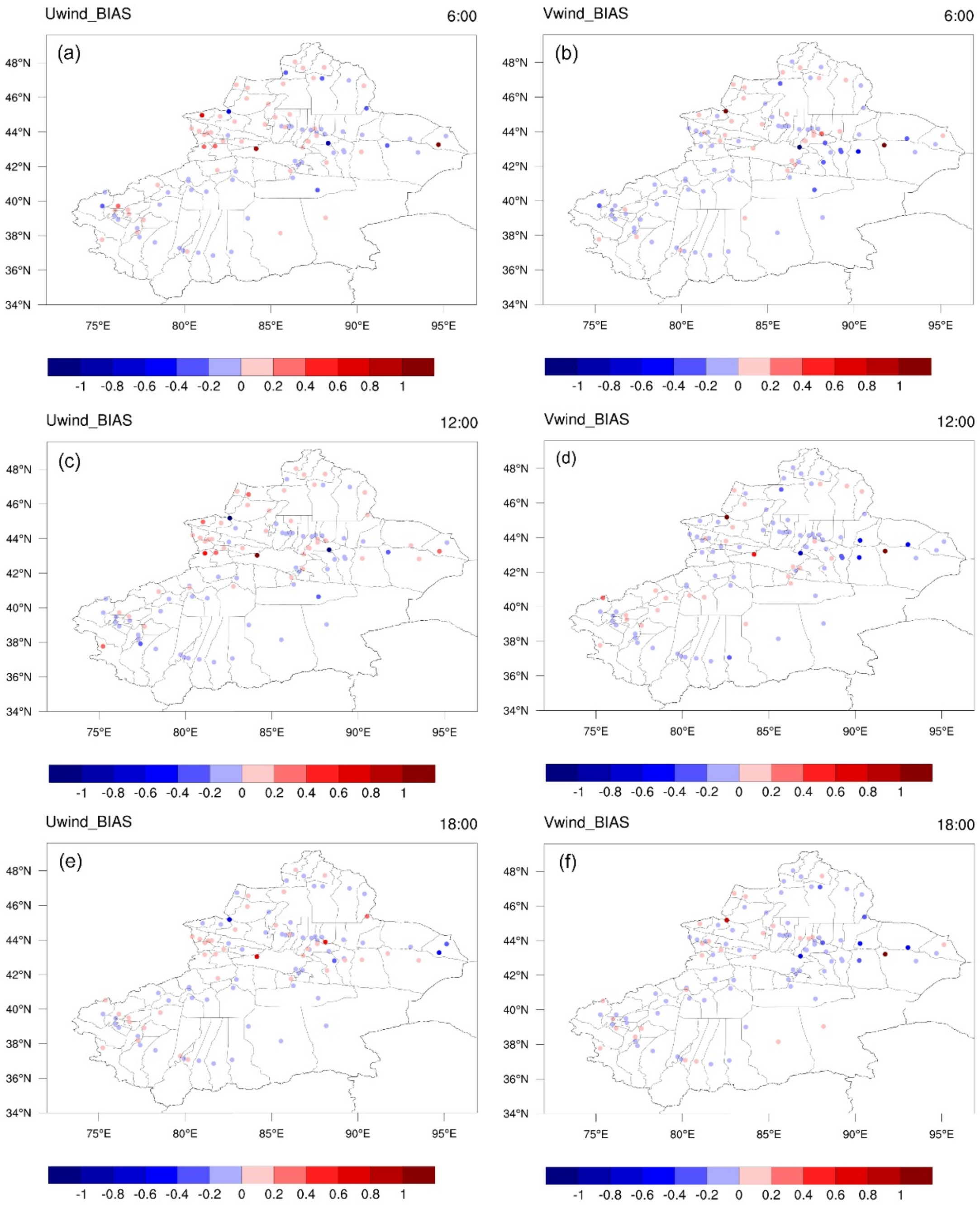

Supplementary Figure S6: Intra-day (6 am-6pm) wind field biases in Xinjiang. The map was generated with NCAR Command Language (Version 6.6.2) [Software]. (2019). Boulder, Colorado: UCAR/NCAR/CISL/TDD. http://dx.doi.org/10.5065/D6WD3XH5.

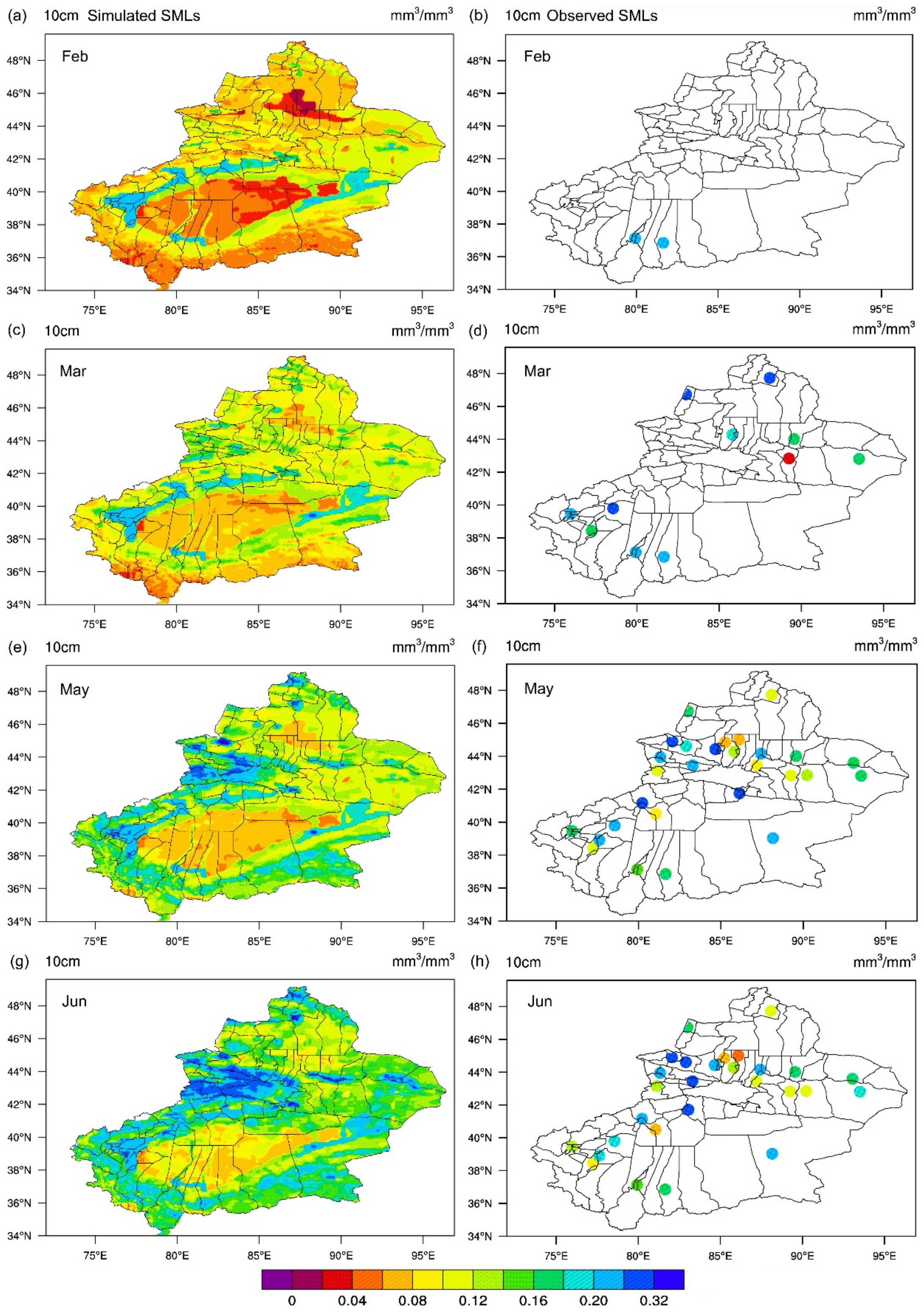

Supplementary Figure S7: Monthly trends for simulated (left panel) versus observed (right panel) SMLs of the 0–10 cm soil layer on Feb, Mar, May and Jun in 2012 (unit: mm^3/mm^3). The map was generated with NCAR Command Language (Version 6.6.2) [Software]. (2019). Boulder, Colorado: UCAR/NCAR/CISL/TDD. http://dx.doi.org/10.5065/D6WD3XH5.

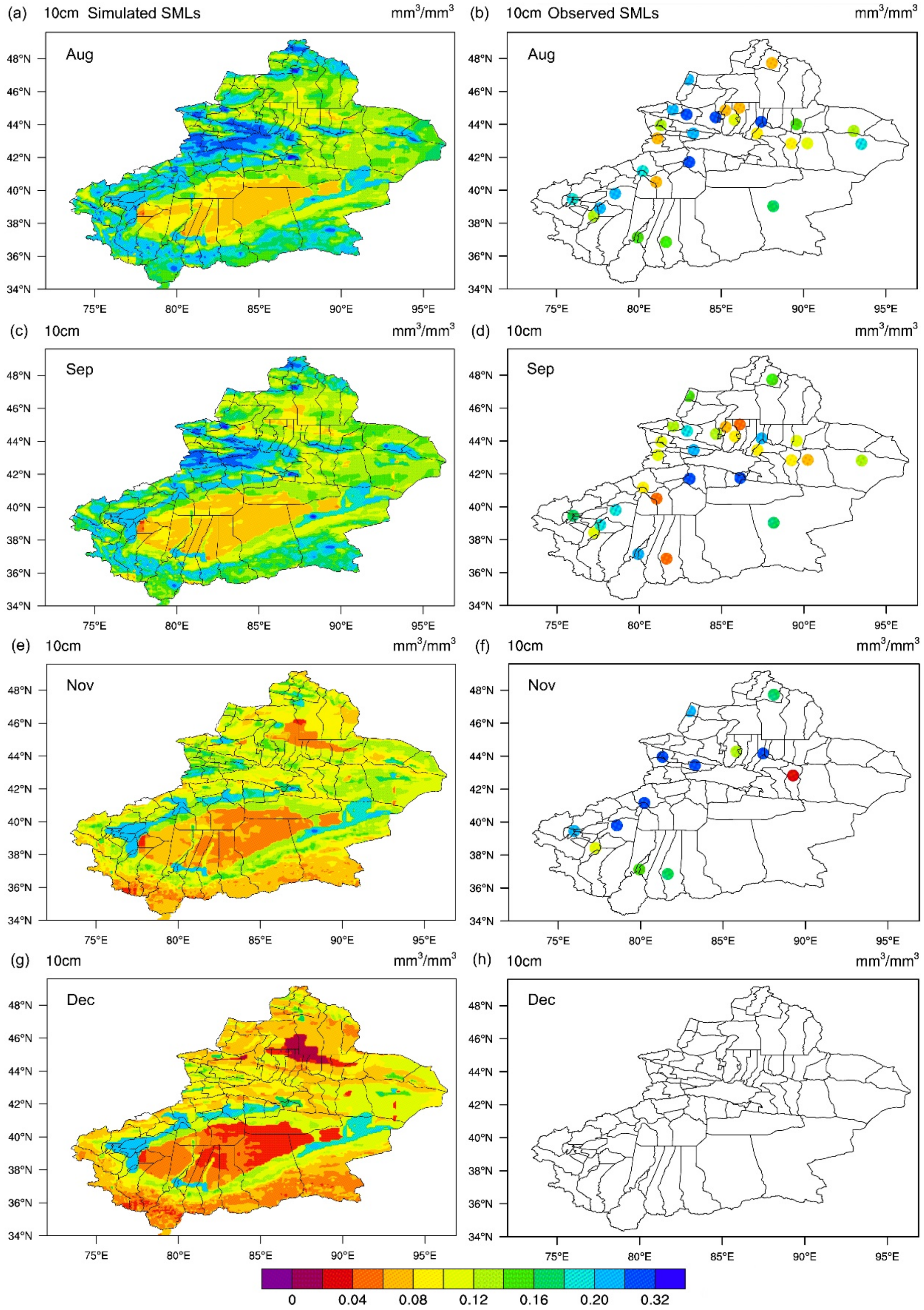

Supplementary Figure S8: Monthly trends for simulated (left panel) versus observed (right panel) SMLs of the 0–10 cm soil layer on Aug, Sep, Nov and Dec in 2012 (unit: mm^3/mm^3). The map was generated with NCAR Command Language (Version 6.6.2) [Software]. (2019). Boulder, Colorado: UCAR/NCAR/CISL/TDD. http://dx.doi.org/10.5065/D6WD3XH5.

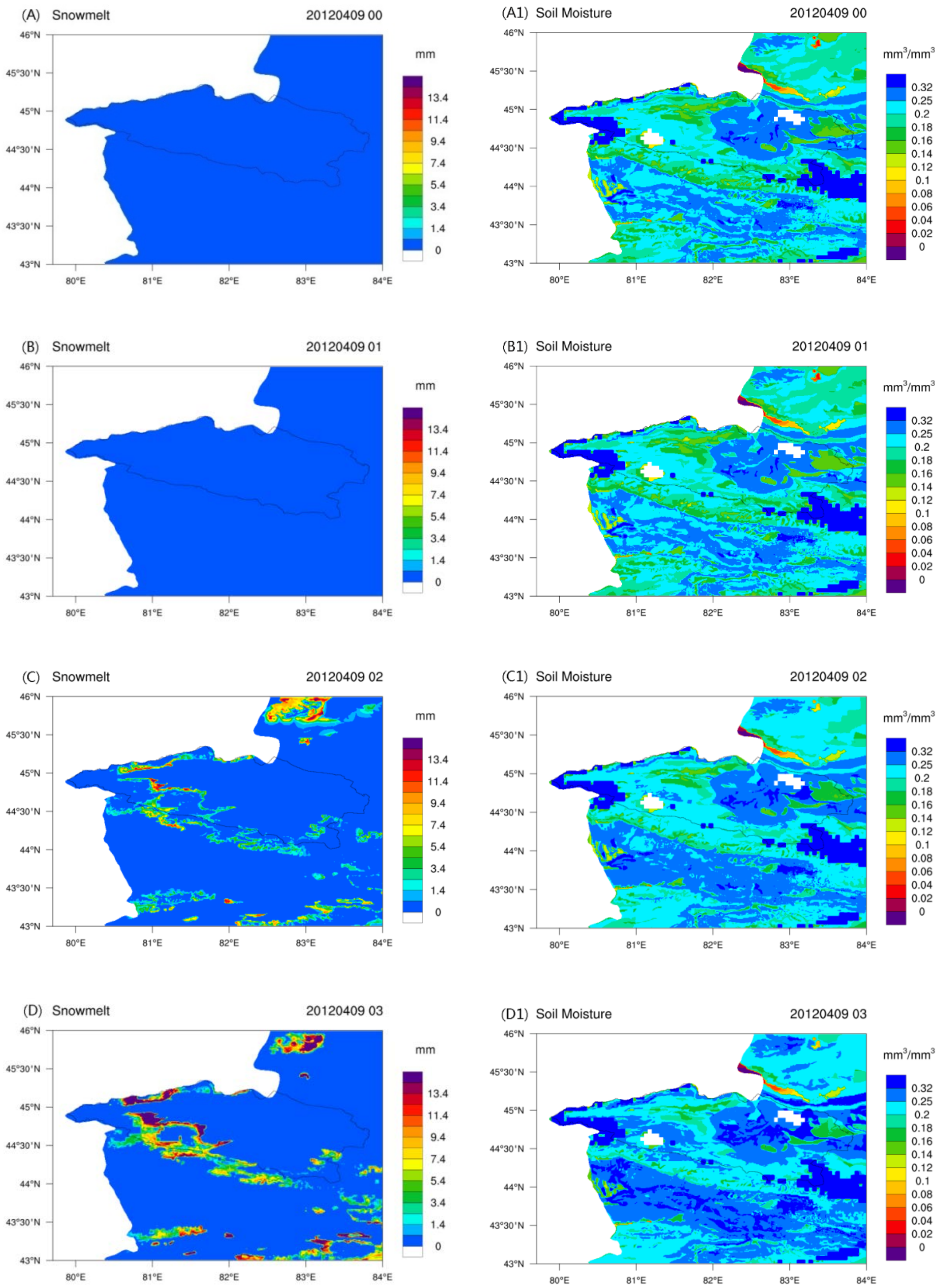
(A) Snowmelt
20120409 00
(A1) Soil Moisture
20120409 00
(B) Snowmelt
20120409 01
(B1) Soil Moisture
20120409 01
(C) Snowmelt
20120409 02
(C1) Soil Moisture
20120409 02
(D) Snowmelt
20120409 03
(D1) Soil Moisture
20120409 03
mm
13.4
11.4
9.4
7.4
5.4
3.4
1.4
0
mm3/mm3
0.32
0.25
0.2
0.18
0.16
0.14
0.12
0.1
0.08
0.06
0.04
0.02
0
46°N
45°30'N
45°N
44°30'N
44°N
43°30'N
43°N
80°E
81°E
82°E
83°E
84°E

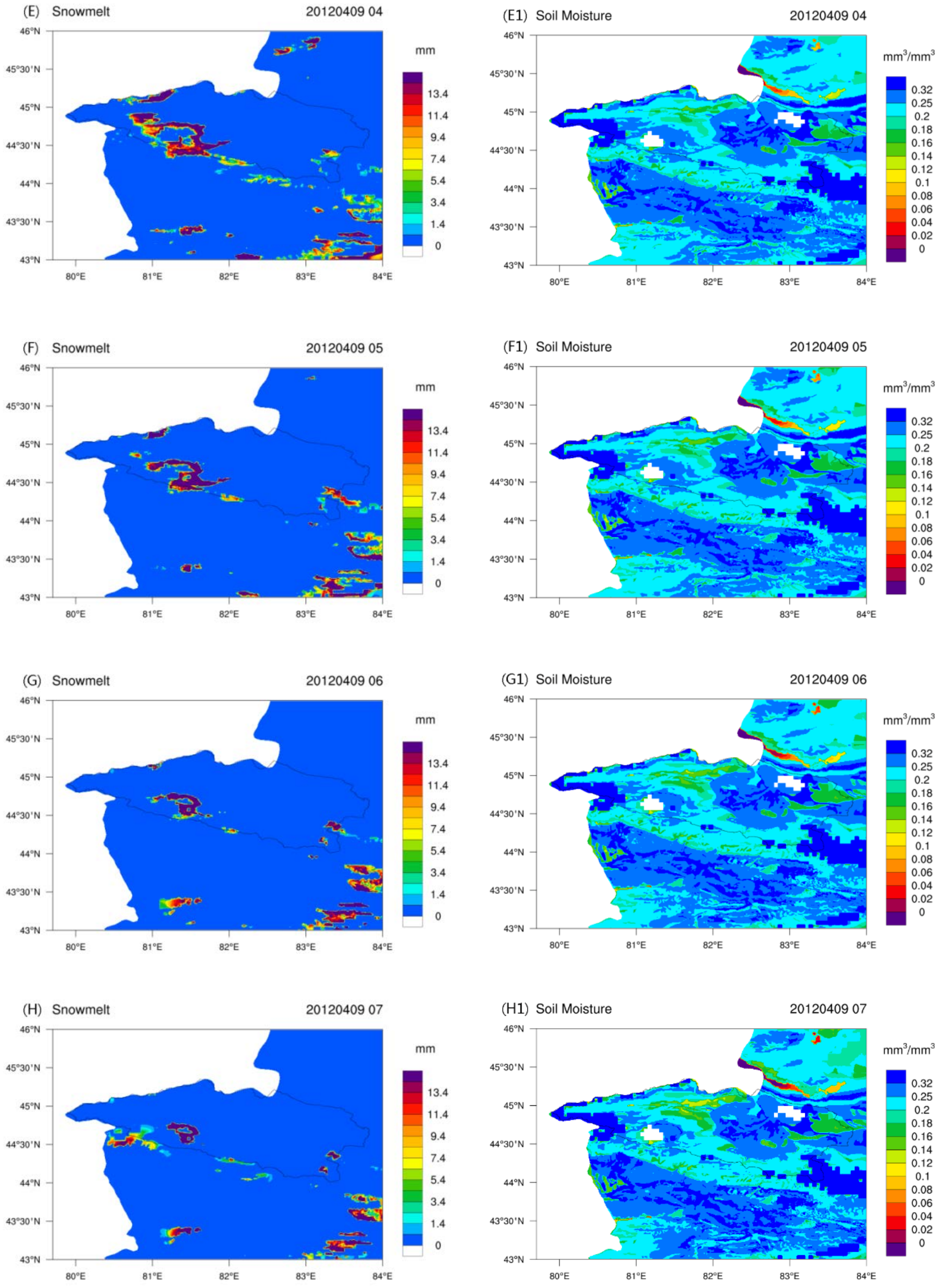
(E) Snowmelt 20120409 04
(E1) Soil Moisture 20120409 04
(F) Snowmelt 20120409 05
(F1) Soil Moisture 20120409 05
(G) Snowmelt 20120409 06
(G1) Soil Moisture 20120409 06
(H) Snowmelt 20120409 07
(H1) Soil Moisture 20120409 07
mm
13.4
11.4
9.4
7.4
5.4
3.4
1.4
0
mm3/mm3
0.32
0.25
0.2
0.18
0.16
0.14
0.12
0.1
0.08
0.06
0.04
0.02
0
46°N
45°30'N
45°N
44°30'N
44°N
43°30'N
43°N
80°E
81°E
82°E
83°E
84°E

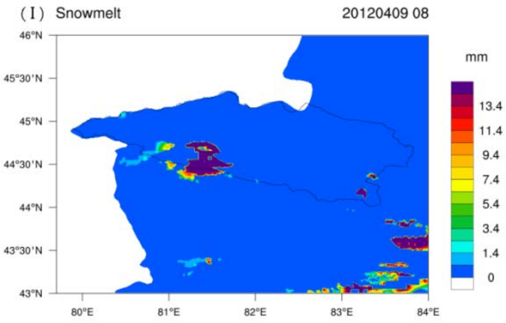
(I) Snowmelt
20120409 08
mm
13.4
11.4
9.4
7.4
5.4
3.4
1.4
0
46°N
45°30'N
45°N
44°30'N
44°N
43°30'N
43°N
80°E
81°E
82°E
83°E
84°E

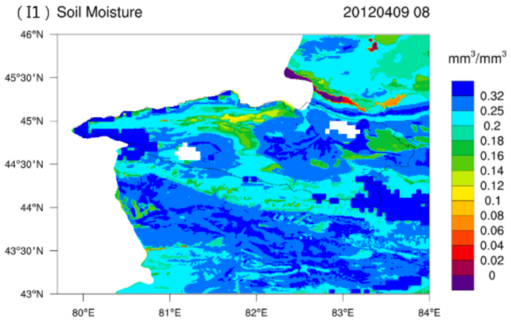
(I1) Soil Moisture
20120409 08
mm3/mm3
0.32
0.25
0.2
0.18
0.16
0.14
0.12
0.1
0.08
0.06
0.04
0.02
0
46°N
45°30'N
45°N
44°30'N
44°N
43°30'N
43°N
80°E
81°E
82°E
83°E
84°E

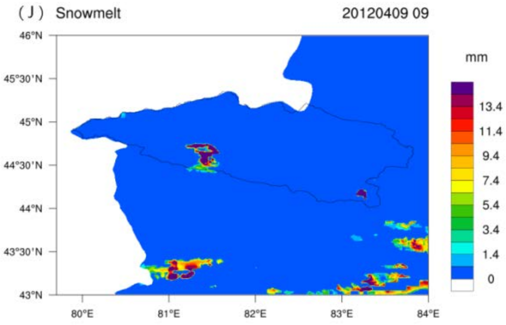
(J) Snowmelt
20120409 09
mm
13.4
11.4
9.4
7.4
5.4
3.4
1.4
0
46°N
45°30'N
45°N
44°30'N
44°N
43°30'N
43°N
80°E
81°E
82°E
83°E
84°E

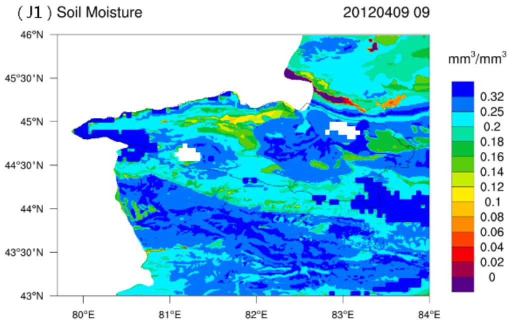
(J1) Soil Moisture
20120409 09
mm3/mm3
0.32
0.25
0.2
0.18
0.16
0.14
0.12
0.1
0.08
0.06
0.04
0.02
0
46°N
45°30'N
45°N
44°30'N
44°N
43°30'N
43°N
80°E
81°E
82°E
83°E
84°E

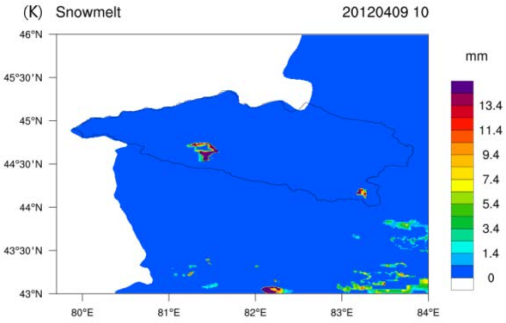
(K) Snowmelt
20120409 10
mm
13.4
11.4
9.4
7.4
5.4
3.4
1.4
0
46°N
45°30'N
45°N
44°30'N
44°N
43°30'N
43°N
80°E
81°E
82°E
83°E
84°E

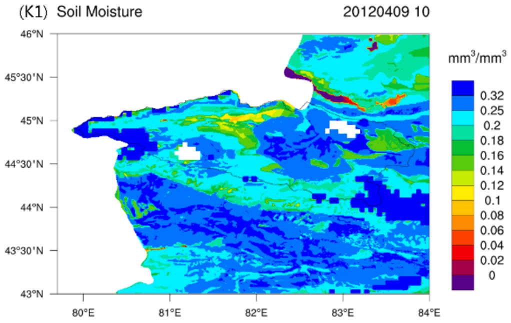
(K1) Soil Moisture
20120409 10
mm3/mm3
0.32
0.25
0.2
0.18
0.16
0.14
0.12
0.1
0.08
0.06
0.04
0.02
0
46°N
45°30'N
45°N
44°30'N
44°N
43°30'N
43°N
80°E
81°E
82°E
83°E
84°E

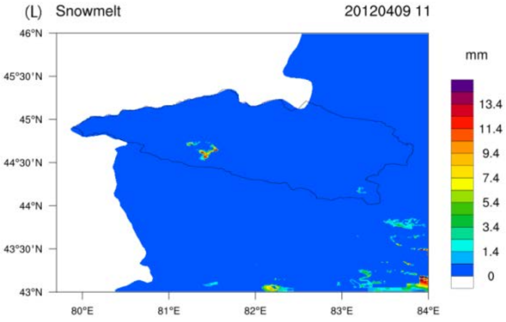
(L) Snowmelt
20120409 11
mm
13.4
11.4
9.4
7.4
5.4
3.4
1.4
0
46°N
45°30'N
45°N
44°30'N
44°N
43°30'N
43°N
80°E
81°E
82°E
83°E
84°E

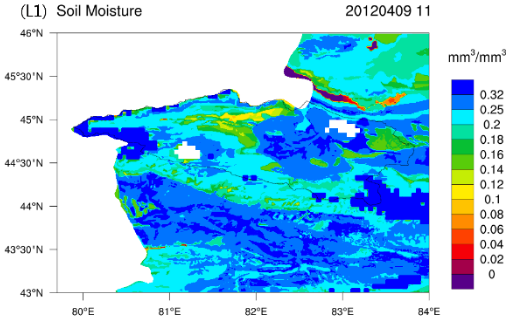
(L1) Soil Moisture
20120409 11
mm3/mm3
0.32
0.25
0.2
0.18
0.16
0.14
0.12
0.1
0.08
0.06
0.04
0.02
0
46°N
45°30'N
45°N
44°30'N
44°N
43°30'N
43°N
80°E
81°E
82°E
83°E
84°E

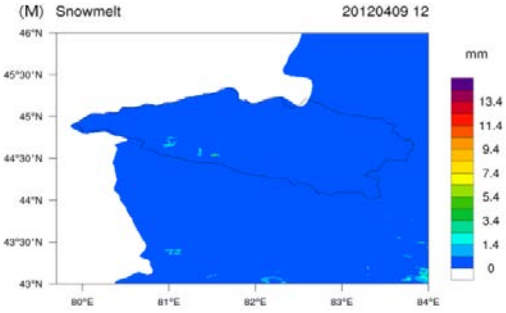
(M) Snowmelt
20120409 12
mm
13.4
11.4
9.4
7.4
5.4
3.4
1.4
0
46°N
45°30'N
45°N
44°30'N
44°N
43°30'N
43°N
80°E
81°E
82°E
83°E
84°E

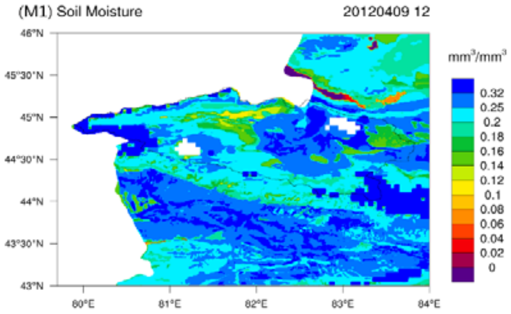
(M1) Soil Moisture
20120409 12
mm3/mm3
0.32
0.25
0.2
0.18
0.16
0.14
0.12
0.1
0.08
0.06
0.04
0.02
0
46°N
45°30'N
45°N
44°30'N
44°N
43°30'N
43°N
80°E
81°E
82°E
83°E
84°E

Supplementary Figure S9: Spatiotemporal distribution of snowmelt in the Bortala–Jing River Basin and the corresponding SML changes. The map was generated with NCAR Command Language (Version 6.6.2) [Software]. (2019). Boulder, Colorado: UCAR/NCAR/CISL/TDD. http://dx.doi.org/10.5065/D6WD3XH5.
